# Relating surface water dynamics in wetlands and lakes to spatial variability in hydrologic signatures

**DOI:** 10.1007/s11273-025-10066-z

**Published:** 2025-07-05

**Authors:** Melanie K. Vanderhoof, Peter Nieuwlandt, Heather E. Golden, Charles R. Lane, Jay R. Christensen, William Keenan, Wayana Dolan

**Affiliations:** U.S. Geological Survey, Geosciences and Environmental Change Science Center, MS 980, Denver Federal Center, PO Box 25046, Denver, CO 80225, USA; Delaware Water Gap National Recreation Area, 1978 River Rd, Bushkill, PA 18324, USA; Office of Research and Development, U.S. Environmental, Protection Agency, 26 W. Martin Luther King Dr., Cincinnati, OH 45268, USA; Office of Research and Development, U.S. Environmental, Protection Agency, 980 College Station Road, Athens, GA 30605, USA; Office of Research and Development, U.S. Environmental, Protection Agency, 26 W. Martin Luther King Dr., Cincinnati, OH 45268, USA; U.S. Geological Survey, Geosciences and Environmental Change Science Center, MS 980, Denver Federal Center, PO Box 25046, Denver, CO 80225, USA; U.S. Geological Survey, Geosciences and Environmental Change Science Center, MS 980, Denver Federal Center, PO Box 25046, Denver, CO 80225, USA

**Keywords:** Floodplain, Flow, Inundation, Lakes, Surface water extent, Stream metrics, Wetlands

## Abstract

The retention of surface water in wetlands and lakes can modify the timing, duration, and magnitude of river discharge. However, efforts to characterize the influence of surface water on discharge regimes have been generally limited to small, wetland-dense watersheds. We developed random forest models to explain spatial variability in six hydrologic signatures, reflecting flashiness, high, and low flow conditions, at 72 gaged watersheds with variable water storage capacity across the conterminous United States. In addition to variables representing meteorology and landscape characteristics, we also tested the inclusion of surface water dynamics, derived from Sentinel-1 and Sentinel-2. Models for all six signatures improved with the addition of catchment characteristics, including surface water dynamics, relative to models with only climate variables. Percent improvement in model adjusted R^2^, mean square error, and Akaike information criterion ranged from 4.00 to 14.33%, 5.00 to 20.30%, and 2.75–8.14, respectively. Automated variable selection can be indicative of the relative importance of certain variables over others. Using a forward selection process, five of the six signature models selected remotely sensed inundation or wetland variables (*p* < 0.05). For example, the variable semi-permanent and permanent (SP + P) floodplain inundation (i.e., lakes along rivers) was associated with lower annual flashiness. Further, SP + P non-floodplain waters and geographically isolated wetlands significantly contributed to explaining variability in the low flow signatures. Our findings underscore the capacity of wetlands to stabilize and maintain flows during dry periods. Improved understanding of how surface water dynamics influence hydrologic signatures can inform wetland restoration efforts and facilitate improved resilience to extreme flow conditions.

## Introduction

Hydroclimatic extremes, which are increasing in frequency, impact both surface water availability and rates of streamflow (Heidari et al. 2020; Wu et al. 2022). Drought events, for example, limit water available for agriculture, drinking water, and wildlife (Stewart et al. 2020; Apurv and Cai 2021), costing the United States $53 billion in just the past five years (2019–2023) (NOAA 2020). Flood events, meanwhile, can endanger property, infrastructure, and human lives, causing global economic damages exceeding $1 trillion between 1980 and 2013 (Winsemius et al. 2016). Surface water storage in wetlands, lakes, and ponds has been shown to increase baseflow and decrease high flows (Rajib et al. 2020; Wu et al. 2020; Zeng et al. 2020). Therefore, accounting for surface water dynamics could improve predictions of hydroclimatic extremes, as well as potentially help mitigate their impacts (Bullock and Acreman 2003; Golden et al. 2021). However, surface water dynamics, despite its potential importance, is still commonly excluded from both hydrological models (Golden et al. 2014; Jones et al. 2019) and analysis of hydrologic signatures (Addor et al. 2018; McMillan 2019).

Hydrologic signatures are quantitative metrics, typically calculated from daily rates of discharge, that describe the magnitude, timing, rate of change, duration, and frequency of flow conditions (Richter et al. 1996; Daigle et al. 2011; McMillan 2019). For example, some signatures reflect wet conditions like flashiness or seasonal flooding (Hannaford and Marsh 2008; Hendry et al. 2019), while others characterize late-season, low flow regimes (Daigle et al. 2011; Kelly and White 2016), or alternatively, the impact of hydrologic alterations, like groundwater pumping, flow diversions, or land use conversions (Richter et al. 1996). The relationship between hydrologic signatures and watershed characteristics, such as climate and topography, has been characterized using statistical techniques including correlation analyses (Berghuijs et al. 2016; Kuentz et al. 2017), random forest models (Trancoso et al. 2016; Addor et al. 2018; Oppel and Schumann 2020) and regression functions (van Dijk 2010; Beck et al. 2015; Kuentz et al. 2017). Generally, hydrologic signatures, in particular high flow signatures (van Dijk 2010; Kuentz et al. 2017), are best explained by climate variables like aridity, precipitation, and snowfall (Beck et al. 2015; Addor et al. 2018). Land cover, such as proportion forest, often acts as a secondary controlling process (Kuentz et al. 2017; Trancoso et al. 2016; Addor et al. 2018). Despite previous findings that surface water influences downstream conditions (Fritz et al. 2018; Lane et al. 2018), efforts to model drivers of hydrologic signatures have rarely included or considered surface water. However, Beck et al. (2013) found the fraction open water, derived from a land cover dataset, showed the greatest variable importance, relative to other climatic and physiographic inputs, in predicting the baseflow index for catchments globally. Expanding the hydrologic signatures tested, Beck et al. (2015) found globally that slope, which can be indicative of potential water storage capacity, outperformed a topographic wetness index and fraction open water and was generally the third highest predictor across signatures, after an aridity index and annual precipitation. Efforts to model drivers of hydrologic signatures have not, to our knowledge, considered the landscape position or hydroperiod of surface water dynamics.

In watersheds lacking surface water storage (e.g., wetlands, lakes, reservoirs, and ponds) when precipitation falls, rain or snow is captured by vegetation, infiltrates the soils, or is transported downgradient as infiltration-excess or saturation-excess runoff (Eamus et al. 2006). Conversely, in watersheds where water storage in wetlands and lakes exists, a portion of the precipitation, snow water equivalent, and runoff can be stored and released through time via groundwater baseflow and fill-spill or fill-merge surface runoff (Rains et al. 2016; Vanderhoof et al. 2016; Stepchinski et al. 2023), with travel time corresponding to watershed position and distance (Fritz et al. 2018; Lane et al. 2018; Wu et al. 2020). For example, as conditions become wetter, non-floodplain wetlands, as well as riparian and floodplain wetlands and lakes, store increased amounts of water, potentially reducing the “flashiness” of river systems (Shaw et al. 2012; Kuppel et al. 2015). During episodic or seasonal highs in discharge, riverbanks can overflow, storing excess water on floodplains and in riparian wetlands, potentially dampening high rates of discharge (Blanchette et al. 2019; Wohl 2021). And, during seasonal low-flow conditions, water in wetlands and other waterbodies percolates through the soil, including between precipitation events, and recharges aquifers, which in turn contribute water to river systems (McLaughlin et al. 2014; Evenson et al. 2015).

Satellites provide a mechanism to track landscape-scale surface water dynamics. For example, cloud-based platforms, like Google Earth Engine (Gorelick et al. 2017), have been used to track global surface water extent from Landsat (Donchyts et al. 2016; Pekel et al. 2016; Pickens et al. 2020). Alternatively, despite its coarser spatial resolution, the short returninterval of the Moderate Resolution Imaging Spectroradiometer (MODIS) has made it appealing for hydrologic applications (Hou et al. 2020; Soulard et al. 2022). Where smaller waterbodies, including wetlands, are of interest, surface water can by tracked using Sentinel-2 (Lefebvre et al. 2019; Ludwig et al. 2019), which provides an improved spatial resolution relative to Landsat and MODIS. As cloud cover presents a major challenge in tracking total surface water over time, SAR satellites like Sentinel-1 can be advantageous, for example to track peak surface water conditions during storm and flood events (Yang et al. 2021). Approaches that use multiple sensors, for instance Sentinel-1 and Sentinel-2, can potentially support higher-frequency observations at finer spatial resolutions (Vanderhoof et al. 2023), which can, in turn, provide improved estimates of surface water dynamics, including hydroperiod.

Hydrologic modeling efforts have demonstrated that potential surface water storage, in select wetland-dense watersheds, can increase baseflow (McLaughlin et al. 2014; Zeng et al. 2020) and reduce high flow and flood duration (Evenson et al. 2018; Ameli and Creed 2019; Wu et al. 2020). However, it is still unclear if the influence of surface water dynamics, particularly relative to the influence of meteorological inputs, can be discerned at greater spatial scales and across watersheds varying in their floodplain and depressional wetland storage capacity. Further research is needed to understand if inundation dynamics within wetlands and lakes show a consistent enough impact to be helpful in modeling discharge regimes across multiple watersheds and regions. To test this, we calculated a suite of hydrologic signatures to characterize the variability in flow flashiness, or the response of streamflow to an event, and flow magnitude, specifically high and low flow conditions, for 72 watersheds across the conterminous United States (CONUS). We developed random forest models for each flow signature: one that considered climate variables only, a second one that considered both climate and catchment variables (e.g., land cover, geology, topography) including variables representing inundation and wetland distribution and hydroperiod, and a third model that considered climate variables and inundation and wetland variables only. The inundation variables were derived from Sentinel-1 and Sentinel-2 surface water algorithms (Vanderhoof et al. 2023). This approach enabled us to assess the relative importance of climate compared to catchment and inundation characteristics. Specifically, our research questions were: (1) What variables help explain the spatial variability in high flow, low flow, and flashiness-related hydrologic signatures? and (2) To what extent do surface water related variables correlate with or help explain the spatial variability in these select hydrologic signatures?

## Materials and Methods

### Watersheds

**Fig. 1:**
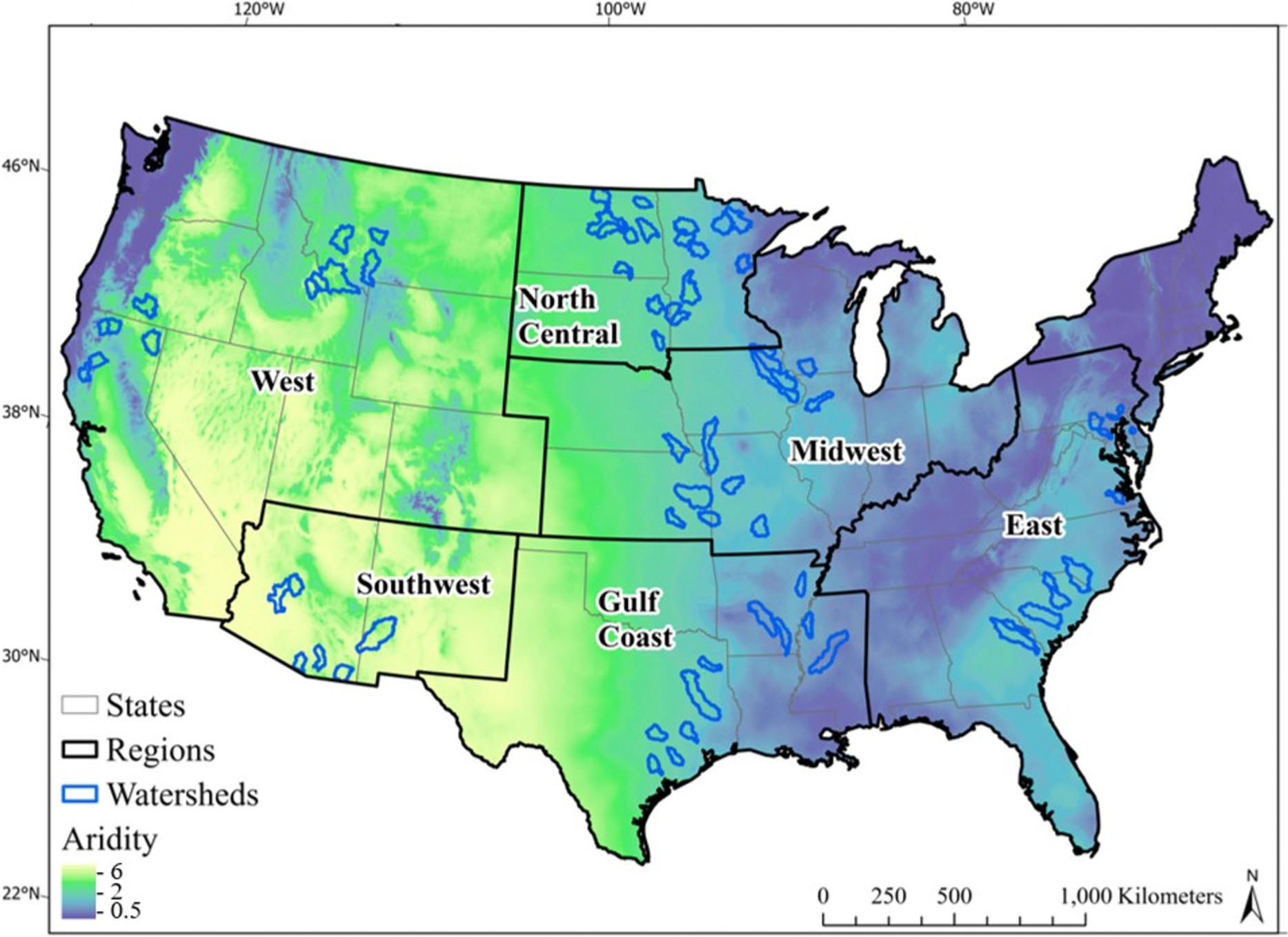
Selected U.S. Geological Survey (USGS) gaged watersheds in relation to aridity (2016–2023), classified into regions. Yellow and purple indicate more and less arid conditions. Legend values show median values for the corresponding colors with a histogram equalize stretch was applied

We selected 72 U.S. Geological Survey (USGS) stream gages and associated watersheds (Fig. 1) across the CONUS from the Geospatial Attributes of Gages for Evaluating Streamflow-II (GAGES-II) dataset (Falcone 2011), representing different land cover types, wetland and river densities, and climate regimes. The GAGES-II dataset is limited to watersheds within the CONUS that are the least disturbed by human activities (Falcone 2011). Watershed size influences both runoff (Pilgrim et al. 1982) and the potential for storage capacity, especially for large waterbodies like lakes. Consequently, we selected non-overlapping watersheds, prioritizing a bounded size class, with 80% between 1500 and 5000 km^2^ (full range = 292 km^2^ to 9918 km^2^). This approach is consistent with the size of 8-digit hydrological units (HUC08, average size = 4374 km^2^), a scale commonly utilized by federal and state agencies for decision-making (Griffith et al. 1999). Gaged watersheds within the Maryland-Delaware area are much smaller than this range, necessitating the inclusion of smaller watersheds in this area. In comparison, most of the Catchment Attributes and Meteorology for Large-sample Studies (CAMELS) watersheds are small (86% < 1500 km^2^) (Newman et al. 2014).

The watersheds were limited to freshwater aquatic systems, excluding watersheds with tidal wetlands. Further, while the avoidance of all dams was not feasible, potential watersheds were reviewed to avoid the inclusion of major dams, defined as dams ≥ 15.2 m in height (storage capacity of ≥ 6.17 MCM) at or near watershed outlets (National Atlas of the United States 2006), which would influence flow dynamics (Rajib et al. 2020), and in turn, the hydrologic signature values. While this selection process limited our watershed sample size, it enhanced our ability to isolate the influence of inundation storage capacity, which depends, in part, on watershed area. We recognize, however, that watershed selection inherently introduces uncertainty (McMillan 2021). For example, while we limited the range of watershed sizes and sampled across multiple regions representing a wide range of aridity conditions, our watersheds were under-sampled in the northeastern U.S. and mountainous regions where a high proportion of forest cover and steep slopes, respectively, tend to increase our uncertainty in mapping surface water. While our watershed sample size was smaller than many studies (e.g., Beck et al. 2015; Kuentz et al. 2017; Addor et al. 2018), it exceeded the gage sample sizes in other analyses of hydrologic signatures (e.g., Oppel and Schumann 2020; Fenicia and McDonnell 2022).

Across the selected watersheds, stream density, as calculated from the National Hydrography Dataset high resolution dataset (NHDplus-HR; USGS 2022), ranged from 259 to 4182 m km^−2^ (median density = 1461 m km^−2^; Table 6). The proportion of each watershed classified as wetland by the National Wetland Inventory (NWI) dataset (USFWS 2019) ranged from 1.1 to 48.7% (median percent wetland = 5.6%; Table 6). Mean annual precipitation (2016–2023) ranged from 325 to 1659 mm (median precipitation = 967 mm; Abatzoglou 2013). Additionally, the dominant landcover class was cultivated crops or hay/pasture for 36 of the watersheds, with other dominant classes including forest (18 watersheds) and grassland-shrub/scrub (13 watersheds) (Homer et al. 2020; Table 6). The watersheds were grouped geographically by U.S. region, including West (n = 12), Southwest (n = 6), North Central (n = 18), Gulf Coast (n = 11), Midwest (n = 13), and East (n = 12), to facilitate data interpretation (Fig. 1).

### Hydrologic signatures: response variables

We calculated hydrologic signatures from daily discharge at each gage, and each signature was a response variable in our statistical analyses (Table 1). Daily rates of stream discharge were acquired from the USGS National Water Information System for 2016–2023 (USGS 2024). While hydrologic signatures can reflect many different aspects of flow (McMillan 2021), including a large number of signatures can make the interpretation of results more difficult. Therefore, signatures were selected from the literature to represent flashiness, or the response of streamflow to an event, and the magnitude of high flows and low flows. We included, (1) the *flashiness index*, which reflects daily variability in discharge across seasons, and was included as a metric on how rapidly streamflow changes in response to snowmelt as well as precipitation events throughout the year (Baker et al. 2004), and (2) *wet season flashiness index*, daily variability in discharge limited to the wet season, defined as the three months in each year with the highest average discharge (Baker et al. 2004). We included two flashiness indices as the flashiness index reflects the response of discharge to episodic events throughout the year including during periods of low flow, while the wet season flashiness is limited to the high flow season. As surface water on the landscape increases during the periods of high flow, the remaining capacity for surface water storage becomes more limited. High flow conditions were further characterized using (3) the maximum annual 30-day flow per drainage area (km^2^) (*MAX30/area*) reflecting seasonal highs in discharge (Hannaford and Marsh 2008); and (4) *(Q10-Q95)/area*, calculated as discharge exceeded 10% of the time, within a given year (Q10, high flows) minus discharge exceeded 95% of the time (Q95, low flows) within a given year, and averaged over multiple years. This metric represents high flows as the average increase in discharge from low flows to high flows (National River Flow Archive 2024). Low flow conditions were characterized using (5) the average driest month discharge per area (*DryMonth/area*, Daigle et al. 2011) and (6) the *baseflow index*, calculated as the ratio of the average annual baseflow volumes to the average annual streamflow volumes (USFS 2022), and (Table 1).

Signatures were either calculated using the calendar year to be unitless or divided by the drainage area (km^2^) so that they could be compared across watersheds (Daigle et al. 2011). We initially explored shorter time scales (i.e., 7-day instead of 30-day) for MAX30/area and DryMonth/area. However, as similar patterns were documented between the two time periods, only the 30-day version was included for both signatures. We also considered including signatures based on the coefficient of variation, but decided they were more challenging to interpret hydrologically, since variability could reflect episodic or seasonal variability. Hydrologic signatures were calculated annually and then averaged across multiple years to reduce uncertainty in the signature values attributable to discharge data errors (Westerberg and McMillan 2015). We used the Shapiro–Wilk test for normality to evaluate the distribution of the hydrologic signature values, and consequently the non-normal indices, flashness index and wet season flashiness index, were normalized using a log10 transform (e.g., Beck et al. 2015).

### Dependence of hydrologic signatures on selected period

The period (2016–2023) over which the signatures were calculated was limited by the temporal availability of Sentinel-2 imagery (Sentinel-2a and −2b launched in June 2015 and March 2017, respectively), required for the surface water algorithms. However, signature uncertainty can increase when using shorter flow records (Kennard et al. 2010). To evaluate potential uncertainty in the hydrologic signature values based on the selected period of analysis, we contrasted the signatures from our 8-year period (2016–2023) with hydrologic signatures derived from a longer 24-year period (2000–2023), using Pearson correlation generated using the Hmsic package in R and relative bias. Between-site variability in the hydrologic signatures derived from the 8-year period, was highly correlated with the between-site variability from a longer, 24-year period (2000–2023) (Table 2). The median value of hydrologic signatures showed some differences between the 8-year period (2016–2023) and the longer 24-year period (2000–2023). Both flashiness indices had a bias of < 1%, but the MAX30/area and (Q10-Q95)/area had a relative bias of 13.5% and 8.7%, respectively, indicating that average high wetness conditions were wetter within the 8-year period, relative to the longer period. Additionally, the DryMonth/area bias was minimal, but the baseflow index showed a relative bias of − 11.8%, potentially reflecting a higher volume of water coming from high flows within the 8-year period, relative to the longer period (Table 2). While the hydrologic signatures of the high and low flow conditions were amplified during the selected period, the signature values between the two periods were highly correlated, with correlation values ranging from 0.94 to 0.99 (Table 2). This suggests that the relative variations in hydrologic signature values between the long-term flow records (24 years) compared to the study period (8 years) are tightly associated.

We also contextualized the study period’s meteorological conditions using the Gridded Surface Meteorological Dataset (GRIDMET, 4 km resolution) 5-day Palmer Drought Severity Index values (PDSI; Abatzoglou 2013). Specifically, we converted PDSI for 1980–2023 to a rank percentile, where 50% represented the median PDSI for the 1980–2023 period. We examined the minimum (i.e., driest), maximum (i.e., wettest) and median per watershed PDSI rank percentile that occurred between 2016 and 2023 to understand the range of PDSI conditions that this 8-year period represents. The 2016–2023 period averaged 5%, 100%, and 62%, for the minimum, maximum, and median PDSI conditions, respectively (Table 6). This indicated that the period was slightly wetter, on average, relative to the longer 44-year period, but that most watersheds exhibited a large range of PDSI conditions (maximum–minimum) over the 2016–2023 period.

### Independent variables

#### Climate variables

The independent variables were used to help predict the hydrologic signatures. To account for potential error in the datasets (Westerberg and McMillan 2015), each climate variable was calculated annually, then averaged over the 2016–2023 study period for each individual watershed. Total annual precipitation and total annual evapotranspiration (ET), using grass as the reference vegetation, were derived from daily GRIDMET (Abatzoglou 2013; Table 3). We also calculated water demand as total annual precipitation – total annual ET (Abatzoglou 2013). An aridity index was calculated as the total annual potential evapotranspiration (PET) divided by the total annual precipitation, where both components were derived from TerraClimate (4.6 km resolution; Abatzoglou et al. 2018; Fig. 1). Higher values represent arid watersheds, and lower values represent less arid watersheds (Budyko 1958). Since only approximately half of the watersheds experience snow, a snowmelt-only variable (e.g., snow water equivalent) was not included. Rather, snowmelt was represented with the variables: a precipitation coefficient of variation (CV), precipitation seasonality, and maximum monthly (January-December) precipitation, all calculated using DAYMET daily precipitation, which includes daily estimates of snow water equivalent (Table 3). Maximum daily temperature was also derived from DAYMET, which has been found to outperform GRIDMET for temperature (Mehdipoor et al. 2018). Temperature variables included maximum temperature seasonality and maximum temperature CV. Both CV variables were calculated from a monthly time step. Precipitation and temperature seasonality were defined as the difference between average summer (June, July, August) and average winter (December, January, February). DAYMET variables relied on 2016–2022 data, as 2023 was not yet available at the time of the analysis.

#### Land cover, soils, topography, and wetland variables

Landscape variables we included for consideration were based on previous efforts that modeled hydrologic signatures (Beck et al. 2015; Trancoso et al. 2016; Kuentz et al. 2017; Addor et al. 2018). Land cover was represented by the 2019 National Land Cover Database (NLCD), as the proportion of each watershed classified as (1) forest (evergreen, deciduous, or mixed), (2) developed (low, medium, and high intensity) and (3) cultivated crops (Homer et al. 2020). To represent soil and geologic characteristics, we included annual minimum depth to water table, depth to bedrock, geologic permeability, fraction clay, fraction silt, and fraction sand from the Soil Survey Geographic Database (SSURGO; Falcone 2011). To represent topography, we used the 10 m USGS Digital Elevation Model (DEM) to calculate the mean percent slope as well as the watershed elevation range divided by its mean elevation (Table 3). We also considered the mean watershed topographic diversity, calculated from the multi-scale Topographic Position Index (mTPI) and the Continuous Heat-Insolation Load Index (CHILI, 30 m resolution; Theobald et al. 2015). We included stream density, which was calculated as the total stream length, defined by the NHDPlus-HR, divided by watershed area (USGS 2022). To represent wetlands, The NWI dataset (USFWS 2019), excluding riverine wetland type, was used to calculate the percent of each watershed mapped as wetlands. We also included the percent of each watershed classified as within the 100-year floodplain (Woznicki et al. 2019). Lastly, the degree of connectivity wetlands have to streams can influence the magnitude and timing of water moving into the stream network. We calculated the percent of each watershed and each watershed’s NWI wetland area that was classified as geographically isolated wetlands (GIWs), as mapped by Lane and D’Amico (2016). GIWs were defined as wetlands that are surrounded by upland and that generally have limited surface water connectivity to downstream waters (Leibowitz 2003).

#### Inundation variables

In addition to including static surface water variables, such as wetland area, remote sensing platforms allow us to include variables that characterize the hydroperiod of surface water stored within watersheds, including wetlands, lakes, ponds, and temporary inundation in flood prone areas. Although Landsat can provide a longer temporal record of surface water dynamics (1984-present), observations are limited to periods free of clouds, snow, and ice, which reduces the accuracy of temporary and seasonal patterns of inundation. Alternatively, the more frequent Sentinel-2 revisit times, and incorporation of a synthetic aperture radar (SAR) satellite like Sentinel-1, can help overcome these limitations. For this analysis we utilized Sentinel-1 and Sentinel-2 based algorithms that were previously developed using gradient boosted classifier algorithms in select regions across the CONUS in Google Earth Engine (20 m resolution; Gorelick et al. 2017; Vanderhoof et al. 2023). Each algorithm was trained to map three classes: non-water, open water (e.g., lakes, ponds, rivers) and vegetated water (e.g., emergent wetlands, forested/shrub wetlands, riparian corridors). A total of 14,400 training points were developed from near-concurrent Landsat, Sentinel-1 and Sentinel-2 images and were divided equally between the three classes. The Sentinel-1 algorithm relied on VV and VH backscatter, topography, recent precipitation, and an annual Sentinel-2 index. The Sentinel-2 algorithm relied on five spectral indices, topography, as well as meteorology, including evapotranspiration and recent precipitation. Additional details on the surface water algorithms can be found in Vanderhoof et al. (2023).

In this effort individual Sentinel-1 and Sentinel-2 images, collected between January 1, 2016, and December 31, 2023, overlapping each of the gaged watersheds (*n* = 72) were classified into open water, vegetated water, and non-water. Cloud and cloud shadows were masked from Sentinel-2 images. We masked pixels with a cloud probability > 30% using the Sentinel-2 Cloud Probability dataset provided by Copernicus. Cloud shadows were identified as pixels with a near-infrared value of < 0.17 within the predicted cloud shadow area, derived from the cloud probability layer and the mean solar azimuth angle. Cloud shadows were buffered by 5 pixels (Vanderhoof et al. 2023). Sentinel-2 images with snow and ice were excluded from 16 of the 32 sites using site-specific seasonal bounds of persistent snow cover (Vanderhoof et al. 2023) and masking out pixels mapped as snow cover outside of those seasonal ranges by the Moderate Resolution Imaging Spectroradiometer (MODIS) snow cover product (Hall and Riggs 2021).

**Fig. 2:**
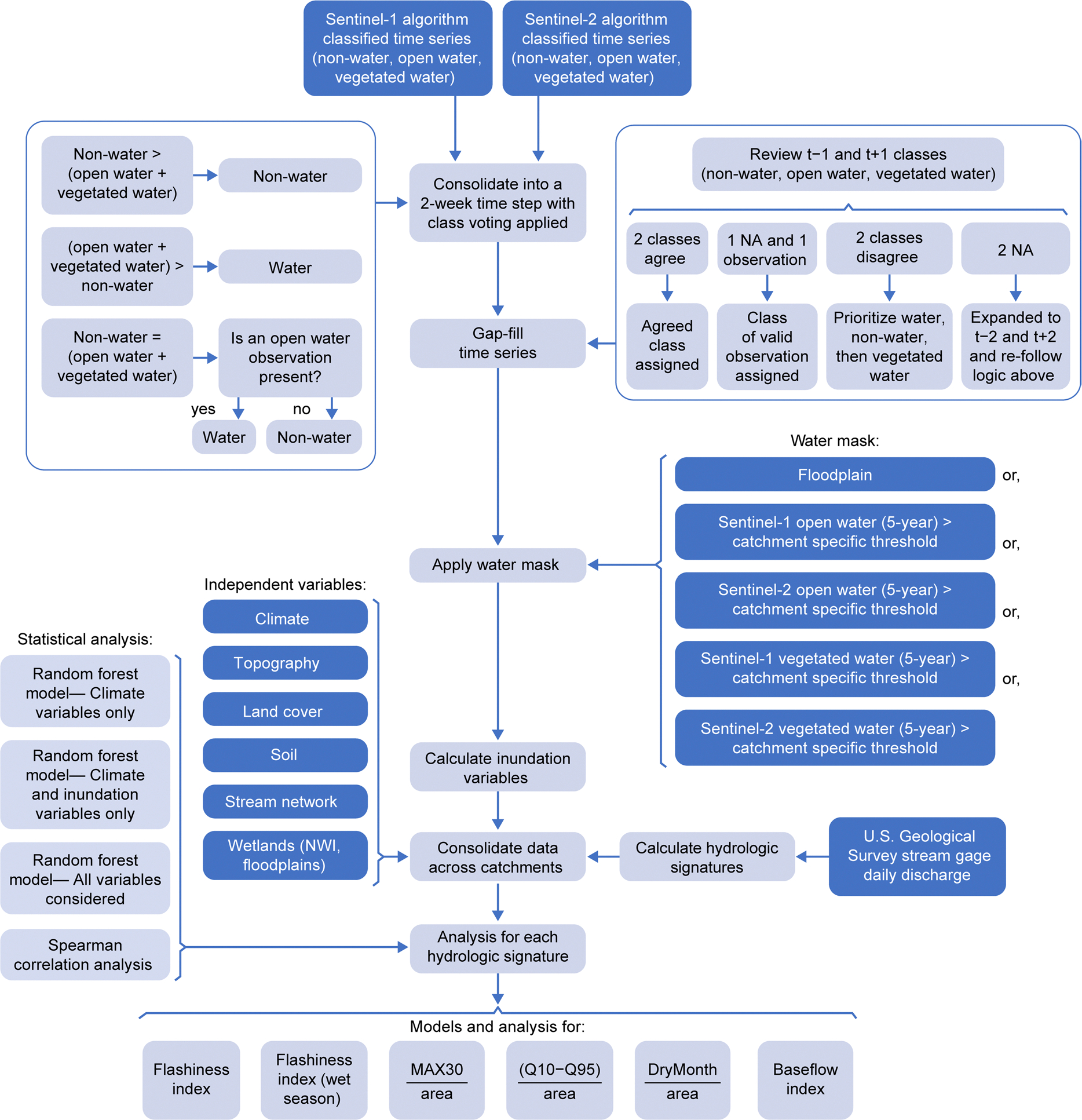
Flowchart of steps to generate the surface water variables and data analysis. Dark blue boxes indicate data inputs

We consolidated the classified Sentinel-1 and classified Sentinel-2 time series at a 14-day time step, compositing an average of two Sentinel-2 images and two Sentinel-1 images for 2017–2021, and two Sentinel-2 images and one Sentinel-1 image for 2022–2023. This composite period minimized data gaps and maximized consistency between Sentinel-1 and Sentinel-2 classifications by improving the probability that both C-band SAR and clear-sky optical data was included in each timestep. We recognize that while the time step allowed for seasonal and interannual patterns in inundation, the influence of episodic events on inundation extent may have been underestimated. Pixel values were assigned as the majority classification, water (defined as open water plus vegetated water), or non-water (Fig. 2). If observations of water and non-water were equal, then we prioritized the open water classification, if present, to improve the probability that high surface water extents were captured. In the absence of an open water classification, we prioritized non-water over vegetated water to reduce commission error (Fig. 2), consistent with the higher accuracy of the open water class relative to the vegetated water class (Vanderhoof et al. 2023). Where no valid observations were present in the 14-day period, we gap-filled pixels using observations from the t−1 and t + 1 timestep, and as needed from t−2 and t + 2 timestep, as shown in Fig. 2.

To limit commission error in the surface water time series and further reduce between sensor classification discrepancies, a water mask, defined as the maximum allowable surface water extent, was derived for each watershed, and applied across the time series. To generate each water mask, we manually reviewed Sentinel-1 open water and vegetated water, and Sentinel-2 open water and vegetated water percentile rasters for each watershed (Fig. 2). We selected percentile thresholds, below which the frequency of erroneously classified water pixels visually exceeded the frequency of correctly classified water pixels (Table 7). We used ancillary data including the NWI dataset (USFWS 2019), the 2019 NLCD (Homer et al. 2020), and base map imagery, delivered through ArcGIS Pro (Redmond CA) to inform the threshold selection. The spatial extent where water pixels were retained was defined as pixels located within the 100year floodplain (Woznicki et al. 2019), to account for short-term flood events, or pixels where the water percentile was greater than the selected threshold in any of the four 5-year percentile rasters (Table 7). Pixels classified as water outside of the water mask were re-classified as non-water.

The Sentinel-1 algorithm has a documented omission (i.e., water was falsely excluded) and commission (i.e., water that was falsely mapped) error of 3.1% and 0.9% for open water, and a 28.4% and 16.0% commission error for vegetated water, respectively, while the Sentinel-2 algorithm has an omission and commission error of 3.1% and 0.5% for open water, and a 10.7% and 7.9% commission error for vegetated water, respectively, when validated against 36 high-resolution images (i.e., WorldView-2, WorldView-3, PlanetScope) (Vanderhoof et al. 2023). When we consolidated Sentinel-1 and Sentinel-2 at a monthly time-step to water and non-water and validated it using 64 PlanetScope images, errors of omission and commission for monthly surface water extent averaged 1.6% and 10.4%, respectively (Vanderhoof et al. 2024a, b). The use of a water mask was previously shown to reduce commission error, resulting in errors of omission and commission of 1.9% and 6.5%, respectively for the monthly surface water extent (Vanderhoof et al. 2024a, b).

**Fig. 3:**
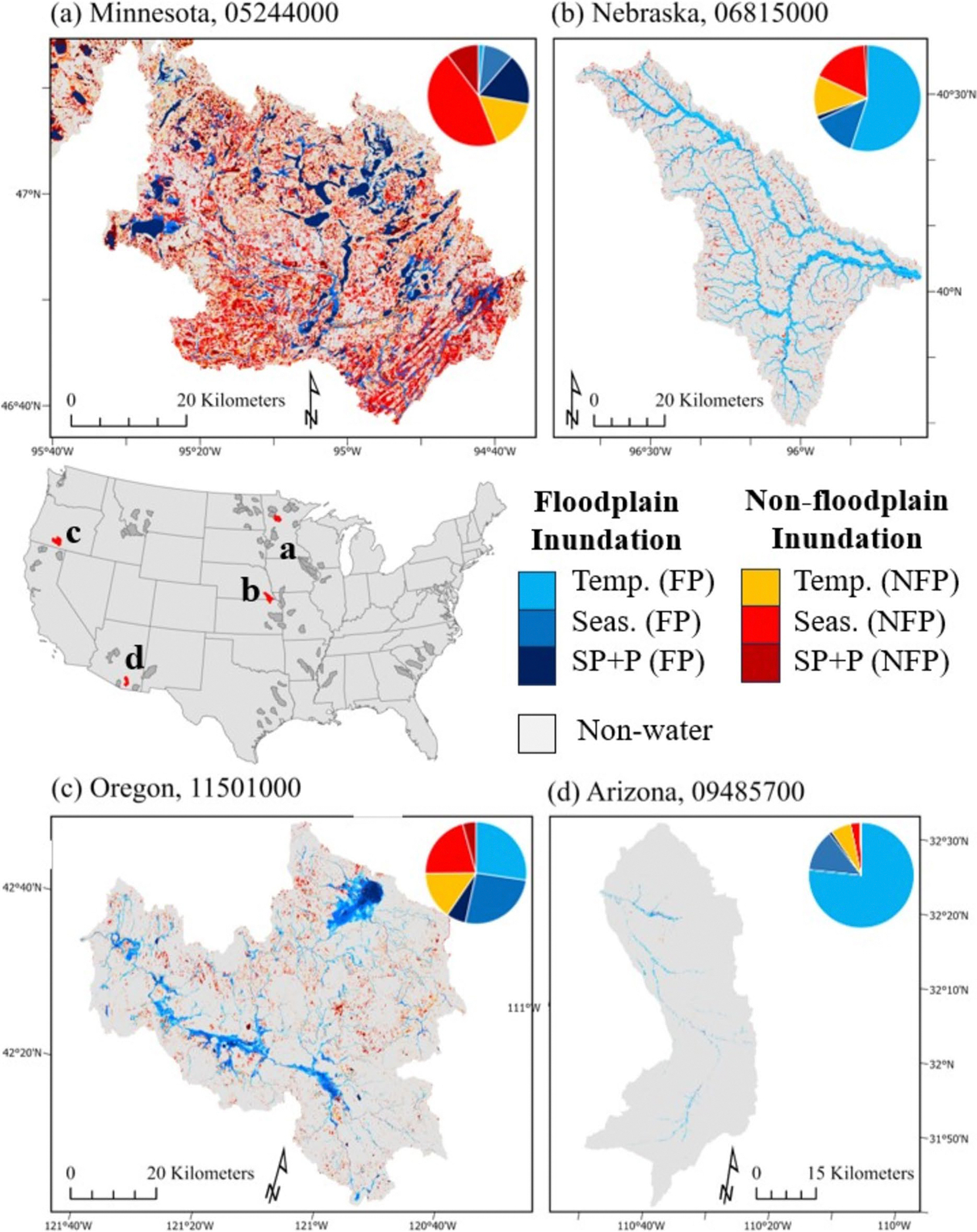
Examples of between watershed variability in the abundance of inundation variables, with the relative distribution of inundation variables shown with pie charts, including **a** MN (gage ID: 05244000), **b** NE (gage ID: 06815000), **c** OR (gage ID: 11501000), **d** AZ (gage ID: 09485700), where the numbers indicate the gage number. *Temp* temporary (> 3 days and < 1 month), *Seas* seasonal (> 1 month and < 6 months), *SP + P* semi-permanent and permanent (> 6 months), *FP* floodplain, *NFP* non-floodplain

After gap-filling and applying the water masks, we consolidated the time series for each watershed into an 8-year percentile. The functional role wetlands impart on downstream discharge reflect the degree and presence of hydrologic connectivity between the two systems. Here we represent connectivity by comparing inundation within and external to the floodplain (Fritz et al. 2018; Lane et al. 2018). Further flow duration and hydroperiod has been identified as critical to quantify water contributions from surface water to the river network (Leibowitz et al. 2018), supporting the characterization of flow regimes (Poff et al. 1997). Therefore, categories of surface water, using the percent of watershed area, were defined in reference to the 100-year floodplain (Woznicki et al. 2019), and included, (1) temporarily flooded, defined as an average of ≥ 3 days but < 1 month per year (Cowardin et al. 1979; Scott et al. 2019), (2) seasonally flooded, defined as inundated > 1 month but < 6 months per year, on average, and (3) semi-permanently and permanently inundated (SP + P), defined as > 6 months per year, on average (Cowardin et al. 1979; Donnelly et al. 2019) (Table 3). We recognize that total surface water extent, particularly in forested environments, is likely to be underestimated by both multispectral and C-band sensors, and that this source of omission potentially impacts the distribution of water by hydroperiod (Huang et al. 2018; Cagnina et al. 2023). L-band SAR data, which can improve efforts to reliably track surface water dynamics under dense canopies (e.g., Hübinger et al. 2024), is limited. Therefore, to help account for this potential seasonal under-estimation of water, we considered water to be SP + P if it was detected for at least 6 of 12 months. The total amount of inundation of any hydroperiod within the 100-year floodplain, and outside of the 100-year floodplain was also included, as was the proportion of inundation that was seasonal (Table 3). Examples of variability in inundation patterns between watersheds are shown in Fig. 3. The terms surface water extent and inundation are used interchangeably in this analysis and represent water that is predominately stored in wetlands and lakes. We recognize that the computationally intensive approach to generate the surface water variables limited our feasible sample size, and consequently likely contributed uncertainty to the modeling effort.

### Modeling analysis

To quantify the correlations between each hydrologic signature and the suite of independent variables we calculated the non-parametric Spearman Rank Correlation Coefficient, generated in R using the Hmisc package. Because of the number of comparisons, a Bonferroni correction was applied before significance was determined (Emerson 2020). We consequently modeled the relationships between each hydrologic signature and multiple predictor variables using random forest regression models developed using the Scikit-learn python package (Pedregosa et al. 2011). For each hydrologic signature, we generated random forest models that considered (1) the inclusion of climate-related variables only ($M_{Climate}$), and (2) the inclusion of all variables, including climate, topography, soil and geology, land cover, and inundation and wetland variables ($M_{\text{All}}$) (Table 3). To further isolate the improvement of the inundation and wetland variables, we also generated an intermediate model that included climate and wetland and inundation variables ($M_{Climate+Water}$). The multi-model approach furthered our ability to quantify the relative contribution of different variable types to explain spatial variability in the hydrologic signatures.

Random forest models use a bootstrapping approach to generate hundreds of regression trees and make no prior assumptions about cause-and-effect relationships or correlations among variables (Hastie et al. 2009). They have also been previously used in the analysis of hydrologic signatures (e.g., Trancoso et al. 2016; Addor et al. 2018; Oppel and Schumann 2020). While random forest techniques are generally insensitive to multicollinearity, the inclusion of highly correlated variables can deflate or bias variable importance values, and complicate model interpretation, making it more challenging to identify the most predictive variables (Murphy et al. 2010; Gregorutti et al. 2016). Conversely, an automated variable selection can be indicative of the relative importance of certain variables over others (Murphy et al. 2010). A stepwise forward selection routine was implemented where the set of potential predictors were sequentially tested. The predictor that contributed most to reducing the root mean square error (RMSE) was selected. During each step, the remaining predictors were removed if they had a correlation value of ≥ 0.85 with any of the selected predictors. This process was iterated until the improvement in the model’s RMSE was < 0.001 with any additional variables, and the variables not selected for inclusion were removed (Sherrouse and Hawbaker 2023). To minimize modeling-related uncertainty, for each model the variable selection and hyperparameter selection processes were concurrently run, where the potential models were compared using a nested cross-validation, KFold with 6 splits (Cawley and Talbot 2010). The hyperparameters tested were n_estimators (the number of trees in the forest with tested values of 300, 500, 700, and 1000) and max_depth (the maximum depth of a tree with tested values of 2, 3, and 4) to limit model overfitting. For all models, max_features (the number of features to consider when looking for the best split) was set at the square root of the number of features, and max_samples (the proportion of samples selected to train each estimator) was set at 0.8. The model with the highest cross-validated adjusted R^2^ was selected.

Random forest models do not consider the spatial pattern between samples, therefore any clustering of the watersheds included in the analysis could potentially bias model predictions (Hengl et al. 2018). Consequently, we tested the residuals of each selected model for spatial autocorrelation using Moran’s I (Klute et al. 2002). Of the random forest model residuals, 6 of 18 showed significant (*p* < 0.05) spatial autocorrelation, the (Q10-Q95)/area ($M_{\text{All}},\;M_{Climate+Water}$), DryMonth/area ($M_{Climate},\;M_{Climate+Water},\;M_{\text{All}}$_),_ and Baseflow index ($M_{\text{All}}$). An autocovariate, or additional model term, representing the mean neighborhood (defined as within 500 km of the catchment center, reflecting catchment clusters) model residual value, was included in these models to account for spatial dependency (Betts et al. 2006).

Performance of the final random forest models was evaluated using the leave-one-out cross validation to account for the limited sample size (*n* = 72) (Vabalas et al. 2019). We calculated the RMSE, R^2^, and adjusted R^2^, to account for differences in the number of variables selected, as well as the Mean Square Error (MSE) and Akaike information criterion (AIC), which were calculated from the observed and model predicted values. Decreases in MSE and AIC indicate model improvement (Portet 2020). Improvement with the inclusion of all variables ($M_{\text{All}}$), relative to $M_{Climate}$, was calculated as the percent change in each performance metric. The percent improvement attributable to inundation or wetland variables was defined for each performance metric as:

(1) $$\frac{\left(M_{\text{Climate}+\text{Water}}-\;M_{\text{Climate}}\right)}{\left(M_{\text{All}}-\;M_{\text{Climate}}\right)}\times 100$$

To evaluate regional patterns, the absolute change in residual values between the $M_{\text{Climate}}$ and $M_{\text{All}}$ models for each index and watershed were summarized by region.

Variable importance for the selected variables in the $M_{\text{All}}$ models was calculated with Python Scikit-learn’s permutation importance. We also calculated the Shapley Additive explanation (SHAP) values, a model agnostic method to explain the contributions of each feature toward model accuracy (Lundberg and Lee 2017). Additionally, significance of model selected variables and the corresponding decrease in MSE with the exclusion of each variable was calculated using the rfPermute package in R using 100 repetitions. The term, significant, across the analysis, was defined as *p* < 0.05 unless specified otherwise.

## Results

### Flashiness signatures

**Fig. 4:**
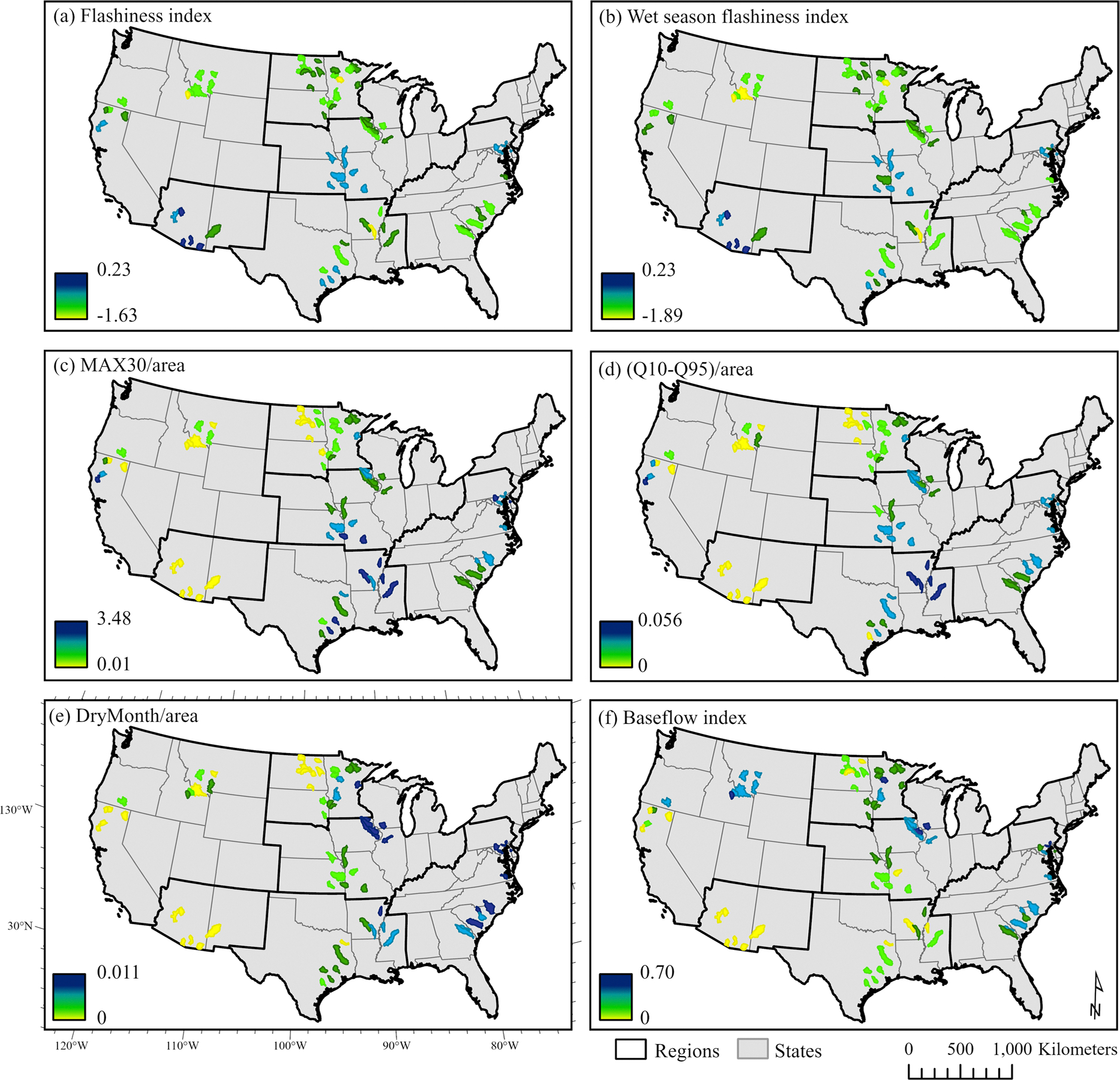
Hydrological signature values by watershed including **a** flashiness index, **b** wet season flashiness index, **c** MAX30/area (m^3^/sec/km^2^), **d** (Q10-Q95)/area (m^3^/sec/km^2^), **e** DryMonth/area (m^3^/sec/km^2^), and **f** baseflow index. Greater flashiness (**a**, **b**), higher high flows (**c**, **d**), and greater flows during low flow periods (**e**, **f**) are shown in blue

**Fig. 5:**
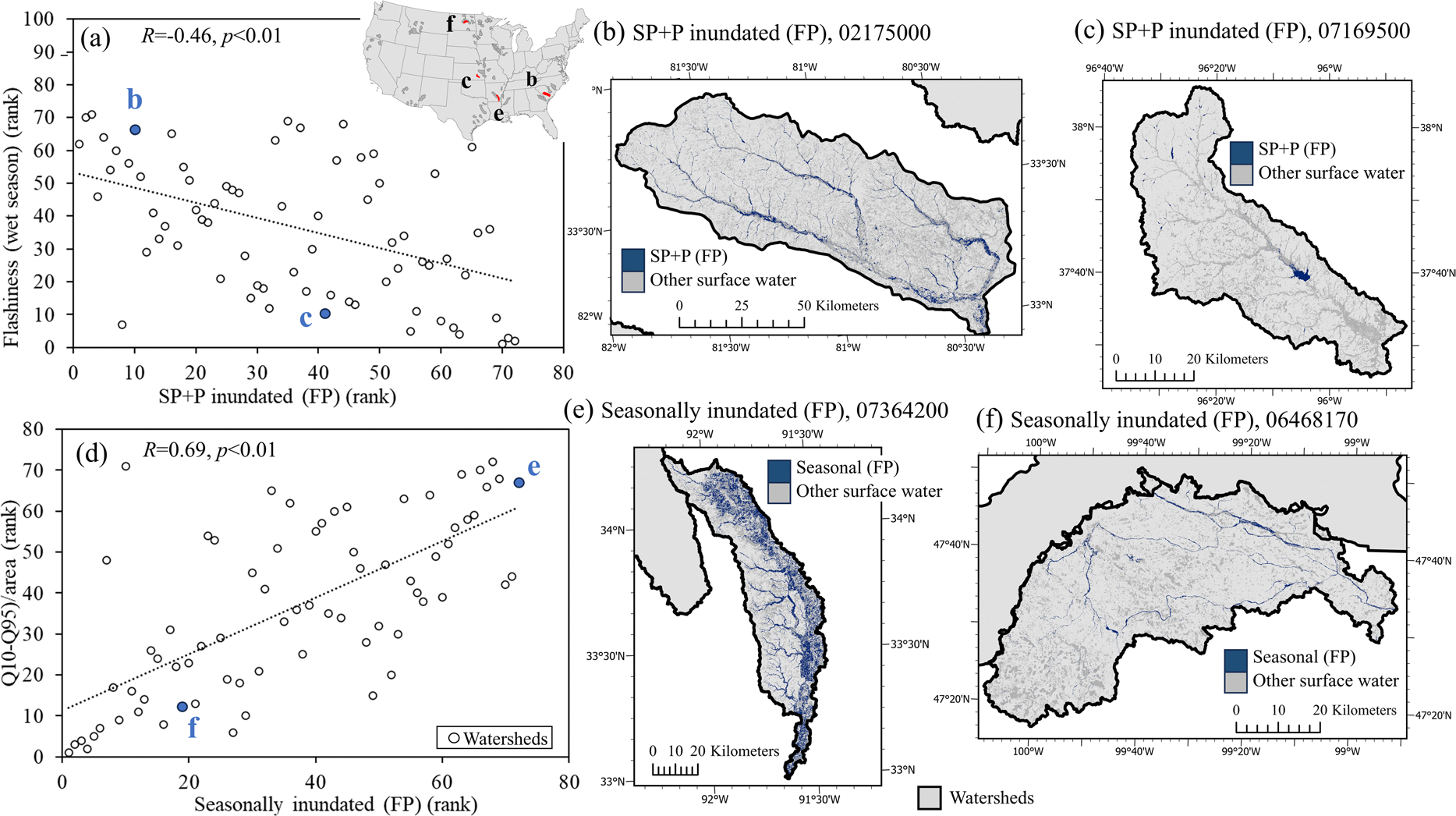
Scatter plot of **a** wet season fashiness versus the percent of semi-permanent and permanent (SP + P) foodplain (FP) inundation, which was included in the $M_{\text{All}}$, with corresponding examples representing examples of the range in SP + P FP inundation (**b, c**) and **d** (Q10-Q95)/area in relation to the percent of seasonally inundated (FP), with corresponding examples showing the range in seasonally inundated (FP) (**e, f**). To match the Spearman correlation analysis, both variables in panels **a** and **d** were converted to rank. The 8-digit number represents the gage identifier

**Fig. 6:**
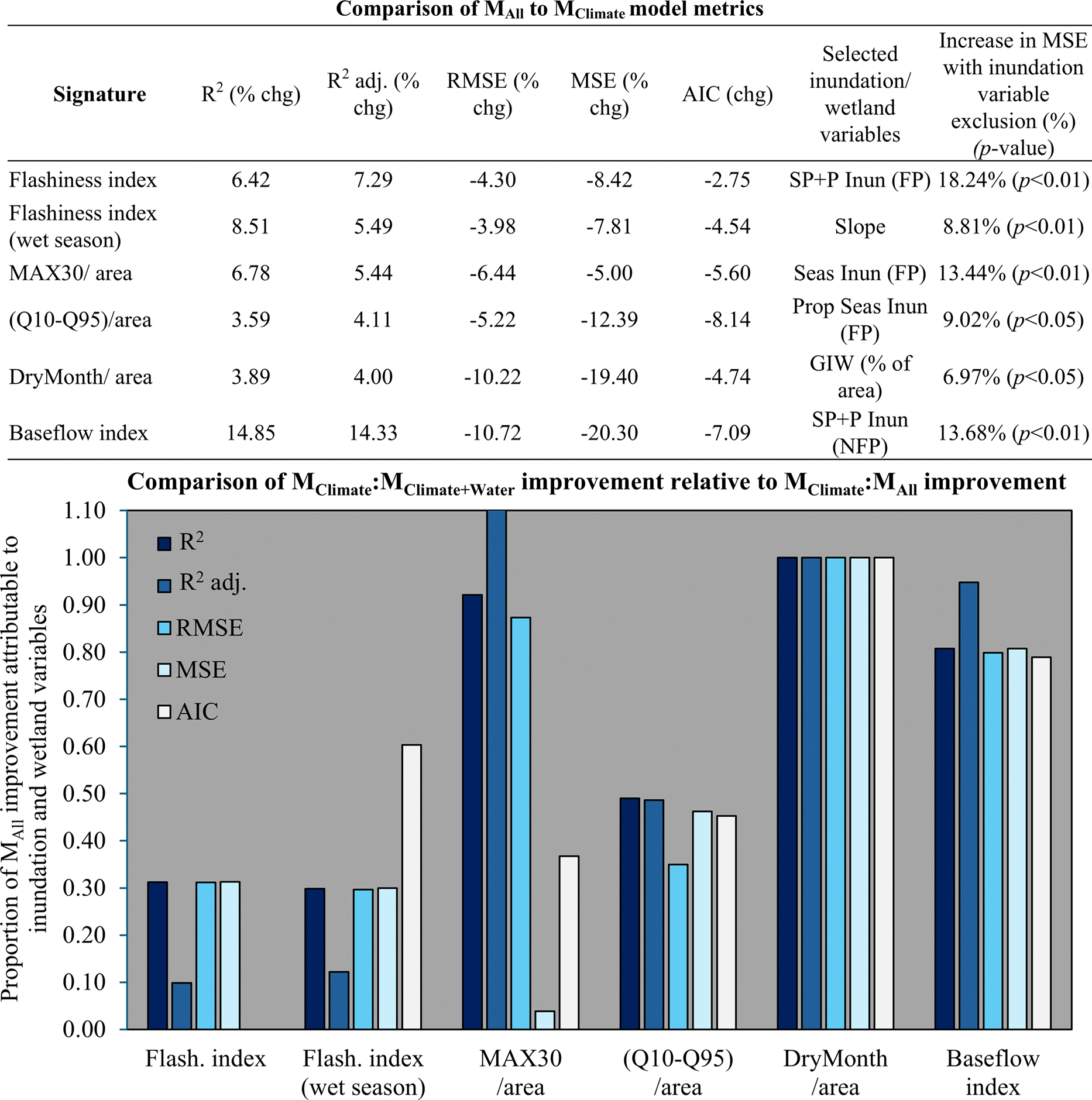
The percent difference in model performance between the $M_{Climate}$ and $M_{\text{All}}$, where positive values for R^2^, and R^2^ adjusted (adj.) indicate model improvement, and negative values for RMSE (root mean square error), MSE (mean square error), and AIC (Akaike information criterion) indicate model improvement. $M_{\text{All}}$ selected inundation and wetland-related variables are also shown. The corresponding bar chart shows the proportion of model improvement, from $M_{Climate}$ to $M_{\text{All}}$, that can be attributed to wetland and inundation variables, using the relative performance of the $M_{Climate+Water}$ models. *MAX* maximum, *chg* change, *SP + P* semi-permanent and permanent inundation, *Seas* seasonally inundated, *FP* floodplain, *NFP* non-floodplain, *Prop* proportion of inundation, *GIW* geographically isolated wetlands

**Fig. 7:**
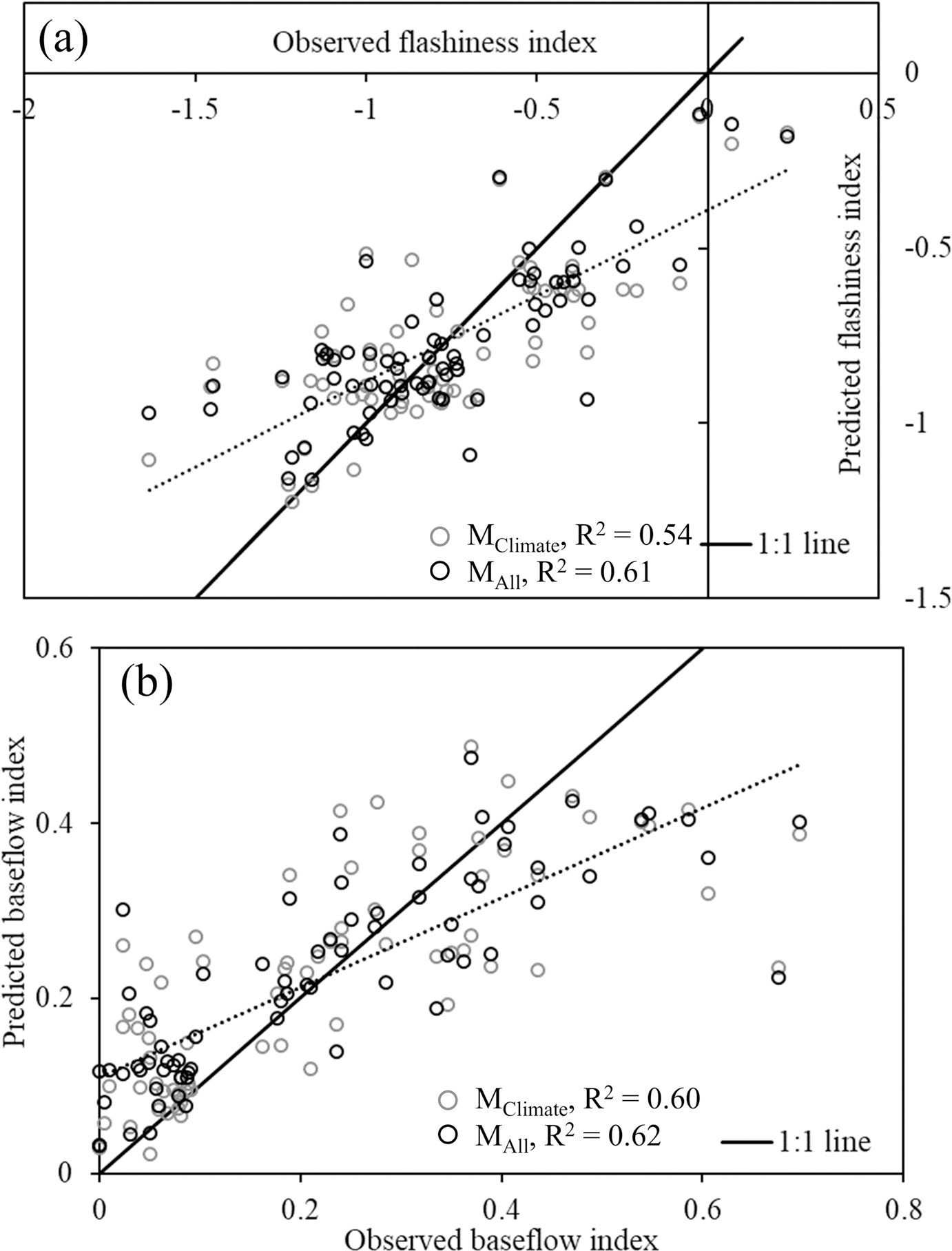
Scatter plots showing observed versus predicted with the $M_{climate}$ and $M_{\text{all}}$ models for a flashiness (unitless) and b baseflow index (unitless)

**Fig. 8:**
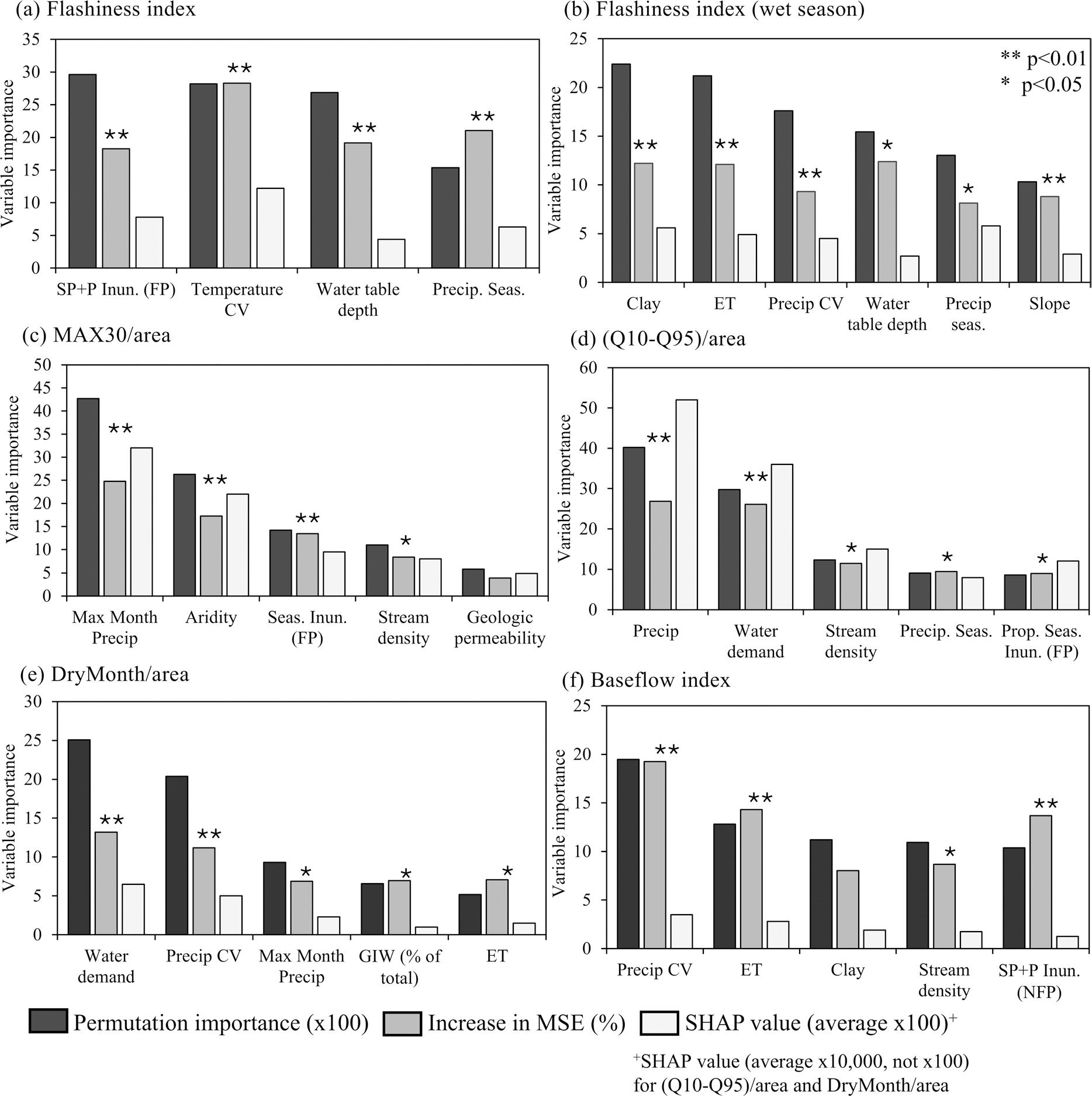
Variable importance for the selected climate and catchment variables within the $M_{\text{All}}$ models, as calculated by permutation importance, increase in mean square error (MSE) with variable exclusion, and SHAP values for **a** Flashiness index, **b** Flashiness index (wet season), **c** MAX30/area, **d** (Q10-Q95)/area, **e** DryMonth/area, and **f** Baseflow index. Stars indicate significance of each variable. Permutation and SHAP values were multiplied by 100 to enhance comparability between importance metrics. *ET* evapotranspiration, *CV* coefficient of variation, *AC* autocovariate, *FP* floodplain, *NFP* non-floodplain, *SP + P* semi-permanent and permanent, *Seas* seasonally, *Inun* inundation, *GIW* geographically isolated wetland, *Q10 and Q95* discharge at 10th and 95th percentiles, *Max* maximum

We found that the flashiness and wet season flashiness indices, were on average highest in the Southwest watersheds, and lowest in the West and North Central watersheds (Table 8, Fig. 4). Watershed flashiness and wet season flashiness were significantly correlated with very few of the independent variables considered. Most prominently, both significantly decreased with greater SP + P floodplain inundation (*R* = − 0.44 and *R* = − 0.46, respectively; Table 4, Fig. 5), and with greater NWI wetland area (*R* = − 0.44 for both, Table 4). Correlations with climate variables were weaker relative to the other hydrologic signatures explored. Despite a high correlation between the two flashiness signatures (*R* = 0.97, *p* < 0.01), the models differed in the variables selected, suggesting that drivers of flashiness over the course of the year were similar but distinct from drivers within the wet season only, when remaining surface water storage may be more limited. The flashiness and wet season flashiness $M_{\text{All}}$ models both saw improvement in explanatory power and associated decreases in the RMSE, MSE and AIC relative to $M_{Climate}$ (Table 5; Figs. 6, 7a). Adjusted R^2^, for example, improved by 7.29% and 5.42% for the flashiness and wet season flashiness, respectively, while MSE decreased by 8.42% and 7.81%, respectively (Fig. 5). Variability in the flashiness signature was best explained by the temperature coefficient of variation (*p* < 0.01) and the amount of SP + P floodplain inundation (*p* < 0.01), as quantified by the three distinct metrics of feature importance. The inclusion of SP + P floodplain inundation was consistent with the correlation analysis (Table 4) and the exclusion of the inundation variable from $M_{\text{All}}$ had a projected increase in MSE of 18.24% (Table 5). The water table depth and precipitation seasonality were also selected for inclusion in the flashiness $M_{\text{All}}$ (Fig. 8). Inundation and wetland variables, as shown by $M_{Climate+Water}$, explained approximately 30%, on average, of the improvement in model performance metrics between $M_{Climate}$ and $M_{\text{All}}$ (Fig. 6).

**Fig. 9:**
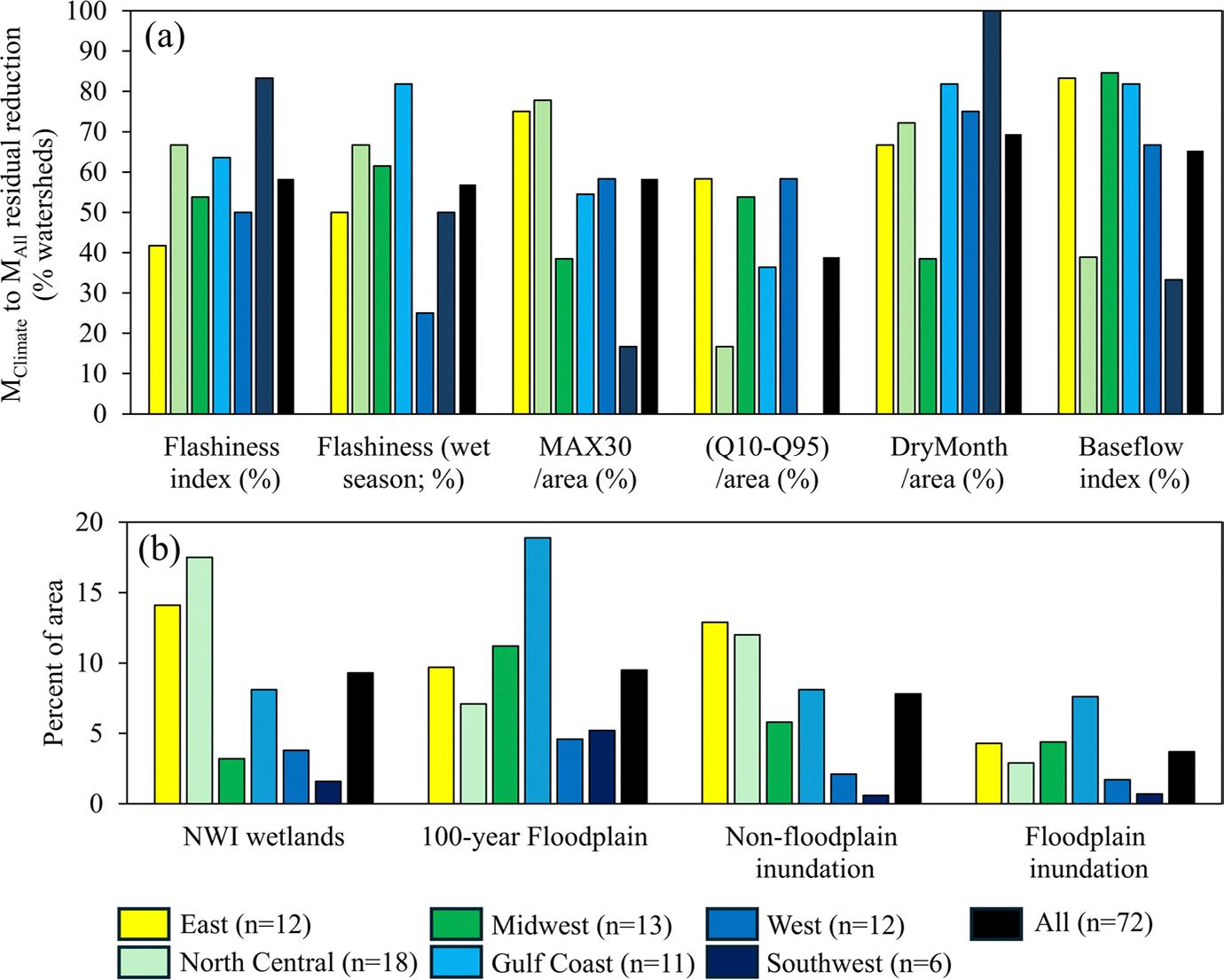
(a) The percent of watersheds within each region that showed a reduction in the absolute model residuals using the $M_{\text{All}}$ model instead of the $M_{Climate}$ model. For comparison, (b) select water variables, averaged across the watersheds within each region, are also shown including wetland area, derived from the National Wetland Inventory, 100-year floodplain, percent of non-floodplain inundation (NFP) and percent of floodplain (FP) inundation, for all hydroperiods

In the wet season flashiness $M_{\text{All}}$_,_ model, the fraction clay and ET had the greatest feature importance (Fig. 8). Fraction clay was negatively correlated with geologic permeability (*R* = − 0.63; Table 10), and positively correlated with the amount of temporary FP inundation (*R* = 0.39; Table 9). Therefore, the clay variable may have more directly reflected maximum potential surface water storage capacity. In addition, slope was also selected for the wet season flashiness $M_{\text{All}}$_,_ model. Slope was negatively correlated with the inundation variable, SP + P floodplain inundation, that was selected for the flashiness $M_{\text{All}}$_,_ model (*R* = − 0.63; Table 9). While no inundation or wetland variables were selected for the wet season flashiness index model, since two of the selected variables, water table depth and slope, were both significantly correlated with many of the inundation variables (maximum correlations with inundation were *R* = 0.71 and *R* = − 0.76, respectively) (Table 9), the $M_{Climate+Water}$ was able to explain 30% of the improvement in three of the model performance metrics and 60% of the AIC improvement observed between the $M_{Climate}$ and $M_{\text{All}}$ models (Fig. 6). Regionally, the $M_{\text{All}}$ models for flashiness and wet season flashiness showed reductions in absolute residual values in wetland dense regions like the North Central (66.7% of watersheds for both signatures) and Gulf Coast (63.6% and 81.8% of watersheds, respectively) but also high flashiness regions like the Southwest (83.3% and 50.0% of watersheds, respectively) (Fig. 9).

### High flow signatures

We found that the high flow signatures, MAX30/area and (Q10-Q95)/area, were highest, on average, within the Gulf Coast watersheds, and lowest, on average, within the Southwest, North Central, and West watersheds, although both signatures saw a high degree of variability across the West region (Table 8, Fig. 4). The two signatures were positively correlated (*R* = 0.93, *p* < 0.01). In relation to the independent variables considered, both signatures, MAX30/area and (Q10-Q95)/area, were most highly positively correlated with precipitation (*R* = 0.86 and *R* = 0.87, respectively), water demand (*R* = 0.78 and *R* = 0.83, respectively), and maximum monthly precipitation (*R* = 0.85 and *R* = 0.82, respectively; Table 4). The high flow signatures were also both significantly correlated with four of the inundation variables. The MAX30/area and (Q10-Q95)/area had a positive, significant correlation with the total amount of floodplain inundation (*R* = 0.60, *R* = 0.63, respectively), the amount of seasonal floodplain inundation (*R* = 0.66 and *R* = 0.69, respectively), and the amount of temporary non-floodplain inundation (*R* = 0.49 and *R* = 0.51, respectively; Table 4). For example, greater (Q10-Q95)/area was correlated with greater amounts of seasonal floodplain inundation (*R* = 0.69, *p* < 0.01; Fig. 5). These correlation values were equivalent to or exceeded correlation with the static water variables, specifically the 100-year floodplain (Table 4).

The MAX30/area and (Q10-Q95)/area $M_{\text{All}}$ models, relative to the $M_{Climate}$ models, showed moderate improvement in explanatory power and associated decreases in the RMSE, MSE and AIC relative to $M_{Climate}$. For instance, the RMSE improved by 6.44% and 5.22%, respectively, and the MSE improved by 5.00% and 12.39%, respectively (Table 5). Consistent with the correlation analysis, maximum monthly precipitation and aridity showed the greatest feature importance for the MAX30/area $M_{\text{All}}$, while total precipitation and water demand had the strongest feature importance for the (Q10-Q95)/area $M_{\text{All}}$ (Fig. 8). Both $M_{\text{All}}$ models selected inundation variables consistent with the correlation analysis, with the MAX30/area $M_{\text{All}}$ selecting seasonal floodplain inundation (*p* < 0.01) and (Q10-Q95)/area $M_{\text{All}}$ selecting the proportion of seasonal floodplain inundation (*p* < 0.05) (Fig. 8). The potential exclusion of the inundation variables had a projected increase in model MSE of 13.44% and 9.02% for MAX30/area and (Q10-Q95)/area, respectively (Table 5). Further, like the flashiness signature, the selected inundation variables (i.e., seasonal floodplain inundation and the proportion seasonal floodplain inundation) were consistent with the inundation variables identified as significant in the correlation analysis (Table 4). In addition, the improvement in model metrics attributable to inundation and wetland variables in $M_{Climate+Water}$ was greater, relative to the flashiness indices, with 66% and 45%, on average across performance metrics, of $M_{\text{All}}$ model improvement, explainable using inundation and wetland variables (Fig. 6). We note that within the MAX30/area $M_{\text{All}}$, while most selected variables were significant, geologic permeability, which was positively correlated with sand fraction (Table 10; *R* = 0.86, *p* < 0.01), was not found to be significant (Fig. 8). By region, the MAX30/area showed the most consistent improvements of residuals in the North Central (77.8% of watersheds) and East (75.0% of watersheds), the regions with the greatest total wetland area. The (Q10-Q95)/area, in contrast showed the most consistent improvements in watersheds within the East, West, and Midwest, where the East and Midwest both contain more floodplain inundation, relative to most other regions (Fig. 9).

### Low flow signatures

We found that the DryMonth/area and baseflow index were highest within the East watersheds, on average, and lowest within the Southwest watersheds (Table 8, Fig. 4). Watersheds were also regionally variable. For example, the DryMonth/area signature graded west (lower) to east (higher) within the North Central region (Fig. 4), concurrent with the aridity gradient within the region (Fig. 1). The two low flow signatures had a significant correlation with one another (*R* = 0.71, *p* < 0.01). However, DryMonth/area was significantly correlated with many more independent variables than the baseflow index. Like the high flow signatures, DryMonth/area was significantly positively correlated with greater annual precipitation (*R* = 0.68) and water demand (*R* = 0.82) and negatively correlated with greater aridity (*R* = −0.86). The DryMonth/area was also significantly positively correlated with total floodplain inundation (*R* = 0.52), seasonal floodplain inundation (*R* = 0.59), and temporary non-floodplain inundation (*R* = 0.58). No significant correlations for DryMonth/area were found with topographic or static wetland variables (Table 4). The baseflow index, in contrast, had relatively few significant correlations, and was negatively significantly correlated with precipitation CV (*R* = − 0.59), evapotranspiration (*R* = − 0.47), and fraction clay (*R* = − 0.44) (Table 4).

Both low flow signatures showed the greatest feature importance for climate variables. Water demand and precipitation CV best explained the DryMonth/area $M_{\text{All}}$, while precipitation CV and evapotranspiration best explained the baseflow index $M_{\text{All}}$. The only non-climate variable selected for inclusion in DryMonth/area $M_{\text{All}}$ was the percent of geographically isolated wetlands (*p* < 0.05), so that 100% of the improvement in model metrics observed in $M_{\text{All}}$ were attributable to inundation and wetland variables (Fig. 6). Within the baseflow index $M_{\text{All}}$, the amount of SP + P non-floodplain inundation (*p* < 0.01), stream density (*p* < 0.05) and fraction clay (not significant) were also selected for inclusion (Fig. 8). Of note, is that the GIW and SP + P non-floodplain inundation variables were also highly correlated with one another (*R* = 0.89, p < 0.01; Table 9). We observed improvement in explanatory power and associated decreases in the RMSE, MSE and AIC in $M_{\text{All}}$ relative to $M_{Climate}$ for both low flow signatures (Table 5). With the inclusion of the geographically isolated wetlands variable, the DryMonth/area v, relative to the $M_{Climate}$, showed a 10.22% and 19.40% improvement in the RMSE and MSE, respectively. Similarly, the baseflow $M_{\text{All}}$, relative to the $M_{Climate}$, showed a 10.72% and 20.30% improvement in the RMSE and MSE, respectively (Fig. 6), with an improved relationship between the observed and predicted baseflow index values (Fig. 7b). For the baseflow index, an average of 83% of the improvement in model performance metrics from $M_{Climate}$ to $M_{\text{All}}$, could be explained by inundation and wetland variables, as shown with $M_{Climate+Water}$ (Fig. 6). Consequently, the DryMonth/area and baseflow index showed not only some of the biggest improvements in model performance from from $M_{Climate}$ to $M_{\text{All}}$, compared to the high flow and flashiness indices, but also the greatest proportion of that improvement that could be explained or attributed to inundation or wetland variables, as shown using $M_{Climate+Water}$. Regionally, the DryMonth/area $M_{\text{All}}$ improved residual values in > 65% of watersheds in all regions except the Midwest, and most consistently in the Gulf Coast, West, and Southwest. The baseflow index $M_{\text{All}}$ improved residual values in > 80% of watersheds in the East, Midwest, and Gulf Coast (Fig. 9).

## Discussion

### Influence of wetland and lake inundation in explaining hydrologic signature variability

The objective of our analysis was to explore whether the influence of wetland and lake surface water dynamics on discharge regimes could be discerned across multiple watersheds with variable discharge characteristics and surface water storage capacity. We found that all six hydrologic signature models improved with the consideration of catchment characteristics ($M_{\text{All}}$), a finding consistent with previous studies (e.g., Beck et al. 2015; Kuentz et al. 2017; Trancoso et al. 2016; Addor et al. 2018). Additionally, all six hydrologic signature models also improved with the inclusion of only inundation and wetland variables ($M_{Climate+Water}$). Each of the six $M_{\text{All}}$ models included only one to three non-climate variables; however, $M_{\text{All}}$ models for four of the six signatures (flashiness index, MAX30/area, (Q10-Q95)/area and baseflow index) selected an inundation variable (significant at *p* < 0.05 within). Of the remaining signatures, the DryMonth/area $M_{\text{All}}$ model included a geographically isolated wetland variable, and the wet season flashiness $M_{\text{All}}$ model selected slope and water table depth variables, which were highly correlated with inundation variables (Table 9). Slope and annual minimum water table depth are, for instance, potential indicators of maximum storage capacity during the wet season, when available storage may already be saturated (Manfreda et al. 2011; Baird and Low 2022). Overall, however, we found improvements in model performance were attributable to both inundation and non-inundation variables. Therefore, it is important to consider how hydrologically meaningful the model improvements were and if the selected inundation variables are consistent with our current understanding of watershed hydrological processes.

The extent of semi-permanent and permanent (SP + P) floodplain inundation showed a high variable importance within the flashiness signature $M_{\text{all}}$ model, where greater SP + P floodplain inundation was associated with lower flashiness, or a lower rate of streamflow rise and fall in response to episodic precipitation and snowmelt events (Hannaford and Marsh 2008). This finding is consistent with stream-connected lakes or large riparian wetlands storing water, delaying the contribution of that stored water to the stream network, and consequently attenuating the rate of daily flow increases and decreases across a watershed (Kuppel et al. 2015; Fritz et al. 2018). Further, improvements in $M_{\text{All}}$ residuals for both flashiness signatures were more consistent across the Gulf coast and North Central, which have a greater abundance of floodplain and wetland area, respectively, relative to the other regions. The variability in high flow signatures, MAX30/area and (Q10-Q95)/area, was explained in part by the amount and proportion of seasonal floodplain inundation, respectively. High flows in our analysis were positively correlated with greater seasonal floodplain inundation. This is consistent with our understanding of seasonal peaks in flow conditions occurring concurrent with seasonal flooding, during which riverbanks overflow storing excess water in floodplain and riparian wetlands (Blanchette et al. 2019; Wohl 2021). We recognize, however, that the positive association between seasonal flooding and high flows in our analysis limits additional insights into the effects of seasonal flooding extent on the timing or magnitude of high discharge conditions. Lastly, SP + P non-floodplain inundation and geographically isolated wetland variables, respectively, were selected and significant for the low flow signature $M_{\text{All}}$ models, the DryMonth/area and baseflow index, respectively. During dry periods, differences in specific yield between uplands and non-floodplain wetlands leads to frequent reversals in hydraulic gradients. This means that non-floodplain wetlands, over time, act as both groundwater sinks (i.e., recharge) and sources (i.e., sub-surface discharge) (McLaughlin et al. 2014), contributing to baseflow (Evenson et al. 2015) and stabilizing low flow conditions (Ameli and Creed 2017; Blanchette et al. 2019). The inclusion of these variables in low flow signature models reflects the potential capacity of non-floodplain and geographically isolated wetlands to support flows during drier periods.

The more frequent model selection of remotely sensed inundation dynamic variables over existing wetland and floodplain dataset variables suggests that consideration of surface water hydroperiod, alongside landscape position, was more helpful in explaining variability in hydrologic signatures than static datasets representing the spatial extent of all wetlands (e.g., NWI) and floodplains (e.g., 100-year floodplain) (e.g., Holt and McMillan 2025). While we recognize that using available datasets (NWI, static floodplains) greatly reduces processing time, dynamic datasets afford improved insights into potential processes influencing stream discharge signatures. Further, while we prioritized a multi-sensor approach to maximize inundation time series consistency as well as the inclusion of vegetated water, inundation variables could also potentially be generated from existing Landsat-based global surface water products (e.g., Pekel et al. 2016; Pickens et al. 2020) to reduce processing time and scale the inclusion of inundation variables.

However, even when incorporating novel, remotely sensed inundation data, characterizing the potential influence of surface water dynamics on river discharge is challenging (Golden et al. 2021). In cases where variables can be isolated (e.g., comparing basins with tile drainage to basins without tile drainage), assessing the differences between two similarly structured models can help quantify the impact of a variable, like tile drainage (Rainio et al. 2024). However, both discharge and surface water extent tend to be a function of climate inputs *and* catchment characteristics (Heimhuber et al. 2016; Vanderhoof et al. 2018). Consequently, our inundation variables were significantly correlated with not only catchment characteristics, such as depth to bedrock, slope, and topographic diversity, but also climate variables, including annual precipitation, aridity, and rainfall intensity (Table 9). The highest correlation, for instance, occurred between the amount of seasonal inundation on the floodplain and annual precipitation (*R* = 0.75, *p* < 0.01) (Table 9). Because our inundation variables were significantly correlated with select climate variables, $M_{Climate}$ cannot be considered a null model, relative to $M_{\text{All}}$. Therefore, evaluating models using the variables selected and their significance and relative importance as well as comparing model improvement using evaluation metrics was seen as more appropriate than testing for significant differences between models. It is possible that an alternative methodological approach, for example integrating inundation data into a process-based hydrologic model (e.g., Stacke and Hagemann 2012; Rajib et al. 2020) or applying a deep learning approach to time series data (e.g., Kratzert et al. 2019), may help build upon these findings and provide additional clarity regarding the discrete role of surface water on diverse discharge regimes. However, process-based hydrologic models on this topic to date have been limited to single watersheds (e.g., Evenson et al. 2018; Ameli and Creed 2019; Jones et al. 2019; Rajib et al. 2020; Zeng et al. 2020), restricting our ability to compare geographically disparate watersheds. Here, we were able to apply and learn from a reduced-form machine learning model to explore the relevance of surface water variables across multiple watersheds, rather than using deterministic process-based modeling approaches that are often only valid in a single watershed.

### Sources of uncertainty

Modeling hydrologic signatures to evaluate the relative influence of climate, catchment, and surface water characteristics on streamflow has multiple potential sources of uncertainty, including the choice of independent and surface water variables, as well as modeling decisions. While we included many climate and catchment characteristics in our models, it is possible that other variables could improve the explanatory power of certain hydrologic signatures. For example, additional data on aquifers (Bloomfield et al. 2021; Holt and McMillan 2025) or geology (Carlier et al. 2018) may help explain more variability in signatures like the baseflow index. Adding novel independent variables could reduce model uncertainty yet the addition of more variables could also decrease model efficiency. For the inundation variables considered, their relative importance within the models may have been influenced by variable error and uncertainty, attributable to the algorithms image classification (Vanderhoof et al. 2023) as well as sensor-related limitations in detecting surface water under dense canopies (Hübinger et al. 2024). It is also possible that while surface water extent was used to represent surface water storage, the conversion of surface water (2D) to storage (3D) could improve its representation in a modeling framework. In the future, potential advancements in satellite-based hydrologic monitoring with sensors like the Surface Water and Ocean Topography (SWOT) and the NASA-ISRO Synthetic Aperture Radar (NISAR) could help improve the representation of surface water storage dynamics.

Though uncertainty reduction is an important goal, our $M_{\text{ALL}}$ models had statistical explanatory power comparable to previous studies. For example, our DryMonth/area model exceeded the modeling performance reported by Addor et al. (2018) and was comparable to Beck et al. (2015). Additionally, our MAX30/area model exceeded the performance reported by Kuentz et al. (2017) but was lower than similar metrics evaluated by Beck et al. (2015). Further, while our baseflow model performance was weaker than that reported by Trancoso et al. (2016), it was comparable to Addor et al. (2018) and Beck et al. (2015) and exceeded values reported by Kuentz et al. (2017). Additionally, we found climate variables, including annual precipitation, evapotranspiration, aridity, and water demand, had the greatest feature importance in most of the $M_{\text{All}}$ models, consistent with the dominant climatic drivers identified by others (Berghujs et al. 2016; Sauqet et al. 2021; Jiang et al. 2022). (Q10-Q95)/area showed relatively minor improvement in some metrics like R^2^ with the inclusion of additional variables including inundation dynamics. This was likely because this signature had relatively high explanatory power using only the $M_{climate}$ models, and therefore had less capacity for improvement, a conclusion consistent with previous studies that have similarly found high flows best predicted by climate variables like precipitation (van Dijk 2010; Kuentz et al. 2017). Further exploration of how inundation may influence aspects of the flow regimes not explored here (e.g., timing, frequency, duration of high and low flow events, and seasonal rising/falling limb rates) will be an important next step to reduce the uncertainty associated with this effort. Future research could investigate how findings may change using other flow signatures or with a sample expanded to include smaller watersheds.

### Management implications

Analysis of signatures related to flow magnitude and variability has applications for managing increases in flood risk (Mogollon et al. 2016), predicting changes in wildlife habitat suitability (Lowe et al. 2019), and evaluating the impact of hydrologic perturbations on vegetation (Richter et al. 1996). Changes in hydrologic signatures over time, in turn, can help quantify the impacts of management actions or evaluate a watershed’s resilience to change (Hannaford and Marsh 2008; Mogollon et al. 2016; McMillan 2021; Lane et al. 2022), where even minor improvements in modeling accuracy can be important for forecasting applications (e.g., Fang et al. 2024). Our analysis, relating watershed characteristics to hydrologic signatures, can be used to inform future watershed management actions. For example, the association of greater SP + P floodplain inundation with less flashiness suggests that protection and restoration of wetlands and lakes within the floodplain and riparian areas may be particularly important to retain and restore in watersheds with flashy discharge. Additionally, we found that non-floodplain surface water inundation contributed to improvements in modeling variability in both low flow signatures, suggesting that the protection and restoration of non-floodplain waterbodies could be prioritized in watersheds where vulnerability to drought has economic consequences. Leveraging satellite-based data sets against streamflow records can advance our ability to support improved watershed management in the face of future floods and drought (Winsemius et al. 2016; Stewart et al. 2020).

## Conclusion

Hydrologic signatures are increasingly used to provide insights on process-based streamflow dynamics. In this analysis, hydrologic signatures provided a mechanism to explore whether inundation dynamics (i.e., extent, watershed position, hydroperiod) within wetlands and lakes improve our capacity to empirically model discharge regimes across multiple watersheds and regions. Adding inundation and wetland variables, either alone or with other catchment characteristics, improved model performance for all six signatures. While climate variables commonly had the greatest variable importance, inundation or wetland-related variables were selected and significant in five of the six hydrologic signature models. Variables representing floodplain inundation, for example, improved predictions of flashiness and high flow signatures. The inclusion of geographically isolated wetlands and semi-permanent and permanent (SP + P) non-floodplain inundation helped explain the spatial variability in low flow signatures (i.e., DryMonth/area and baseflow index, respectively). These low flow signatures had the greatest average improvement in model performance from $M_{Climate}$ to $M_{\text{All}}$, relative to the high flow and flashiness indices. Additionally, much of that improvement was explained or attributable to inundation or wetland variables, as shown using $M_{Climate+Water}$. This finding points to the potential value of low-connectivity wetlands in mitigating drought impacts. Enhancing our understanding of how surface water dynamics influence discharge regimes can help guide management of non-riverine surface waters, including wetlands, lakes, and floodplains, thereby supporting greater watershed resilience against climate extremes and hydrologic disturbances.

## Figures and Tables

**Table 1:** Hydrological signatures included in the analysis. MAX: maximum

Signature	Targeted flow regime	Calculation	Units	Median	Min	Max	Source

Flashiness index	All flows	The sum of the absolute value of the changes in discharge from the day prior to the current day (discharge t_2_—discharge t_1_) divided by the sum of the daily discharge values (log normalized)	Unitless	−0.81	−1.63	0.23	Baker et al. (2004)
Flashiness index (wet season)	High flows	The sum of the absolute value of the changes in discharge from the day prior in the three wettest months (highest discharge) divided by the sum of daily discharge values in those months (log normalized)	Unitless	−0.84	−1.89	0.23	Baker et al. (2004)
MAX30/area	High flows	The flow rate for the 30 days per year with the highest flow rate, summed over the 30 days, and averaged per year, divided by the watershed area	m^3^/sec/km^2^	0.94	0.01	3.48	Hannaford and Marsh (2008)
(Q10-Q95)/area	High flows	Discharge exceeded 10% of the time (Q10) minus discharge exceeded 95% of the time (Q95), divided by watershed area	m^3^/sec/km^2^	0.016	0.000	0.056	National River Flow Archive (2024)
DryMonth/area	Low flows	Average annual discharge in the driest month (excluding snow cover months) divided by watershed area	m^3^/sec/km^2^	0.0019	0.0000	0.0112	Daigle et al. (2011)
Baseflow index	Low flows	The ratio of the average daily flow during the lowest annual 7-day flow (excluding snow cover conditions) to the annual average daily flow	Unitless	0.19	0.00	0.70	USFS (2022)

**Table 2:** Correlation values comparing the 2016–2023 hydrologic signatures with the same signatures derived from the 2000–2023 period

Metric	*R* (2016–2023 vs 2000–2023)	Median relative bias (%)

Flashiness index	0.99	0.9
Flashiness index (wet season)	0.99	0.2
MAX30/area	0.97	13.5
(Q10-Q95)/area	0.98	8.7
DryMonth/area	0.94	−2.2
Baseflow index	0.95	−11.8

The relative bias compares the paired signature values from each watershed. All *R* values were significant at *p* < 0.01

*MAX* maximum

**Table 3:** Independent variables considered modeling hydrological signatures

Variable type	Variable	Units	Min	Max	Median	Source

Climate	Precipitation (P, annual)	mm	325.3	1659.1	967.4	GRIDMET Abatzoglou (2013)
	Evapotranspiration (ET, annual)	mm	714	1934.1	1181.1	GRIDMET Abatzoglou (2013)
	Aridity index (PET/P, annual)	unitless	0.8	6.63	1.21	TerraClimate Abatzoglou et al. (2018)
	Water demand (P—ET, annual)	mm	−1586	265.6	−247.4	GRIDMET Abatzoglou (2013)
	Precipitation seasonality	mm	−396	276.6	105	DAYMET Thornton et al. (2020)
	Precipitation CV	unitless	0.41	1.31	0.64	DAYMET Thornton et al. (2020)
	Maximum monthly precipitation	mm	53.9	230.8	131.6	DAYMET Thornton et al. (2020)
	Temperature seasonality	°C	15.6	34.2	23	DAYMET Thornton et al. (2020)
	Temperature CV	unitless	0.23	1.3	0.48	DAYMET Thornton et al. (2020)
Land Cover	Forest (evergreen, deciduous, mixed)	% of area	0.059	56.1	17.5	NLCD (2019); Homer et al. (2020)
	Developed (low, medium, high intensity, open space)	% of area	0.323	35.7	4.69	NLCD (2019); Homer et al. (2020)
	Cultivated crops	% of area	0.0	84.7	17.9	NLCD (2019); Homer et al. (2020)
	Stream density	m km^2^	259.2	4181.6	1460.9	NHDPlus High Res. USGS (2022)
Soil and Geology	Clay fraction	fraction	0.08	0.47	0.23	SSURGO Falcone (2011)
	Sand fraction	fraction	0.07	0.74	0.33	SSURGO Falcone (2011)
	Silt fraction	fraction	0.17	0.72	0.44	SSURGO Falcone (2011)
	Depth to bedrock	cm	81.3	152.4	145.8	SSURGO Falcone (2011)
	Annual min depth to water table	meters	0.49	1.83	1.40	SSURGO Falcone (2011)
	Geologic permeability	cm day^−1^	0.5	8.7	2.2	SSURGO Falcone (2011)
Topography	Slope	%	0.5	32.5	3.7	DEM Gesch et al. (2002)
	(Elevation_max_—Elevation_min_) / Elevation_average_	unitless	0.2	4.9	1.0	DEM Gesch et al. (2002)
	Global SRTM topographic diversity	unitless	0.03	0.7	0.1	Theobald et al. (2015)
Inundation Dynamics	Temporarily flooded, floodplain (3 days—1 month)	% of area	0.07	4.16	0.65	Vanderhoof et al. (2023)
	Temporarily inundated, non-floodplain (3 days—1 month)	% of area	0.03	5..85	1.29	Vanderhoof et al. (2023)
	Seasonally inundated, floodplain (1—6 month)	% of area	0.04	8.58	1.77	Vanderhoof et al. (2023)
	Seasonally inundated, non-floodplain (1—6 month)	% of area	0.01	45.81	4.07	Vanderhoof et al. (2023)
	Semi-permanently and permanently inundated, floodplain (> 6 month)	% of area	0	3.54	0.39	Vanderhoof et al. (2023)
	Semi-permanently and permanently inundated, non-floodplain (> 6 month)	% of area	0	5.55	0.44	Vanderhoof et al. (2023)
	Total floodplain inundation	% of area	0.42	15.46	3.08	Vanderhoof et al. (2023)
	Total non-floodplain inundation	% of area	0.04	52.59	6.06	Vanderhoof et al. (2023)
	Proportion of inundation that is seasonally inundated, floodplain (1—6 months)	% of inundation	3.15	53.56	18.34	Vanderhoof et al. (2023)
	Proportion of inundation that is seasonally inundated, non-floodplain (1—6 months)	% of inundation	2.16	77.44	39.64	Vanderhoof et al. (2023)
Wetland	Geographically Isolated Wetlands (GIW)	% of area	0.0	9.4	0.6	Lane and D’Amico (2016)
	Proportion of wetland area identified as GIW	% of area	0.6	80.9	11.4	Lane and D’Amico (2016), USFWS (2019)
	Floodplain	% of area	1.2	36.8	7.7	Woznicki, et al. (2019)
	National Wetland Inventory (NWI) wetlands	% of area	1.1	48.7	5.6	NWI USFWS (2019)

*DEM* Digital elevation model, *SRTM* Shuttle Radar Topography Mission, *NLCD* National Land Cover Database, *SSURGO* Soil Survey Geographic Database, *NHD* National Hydrography Dataset, *CV* coefficient of variation, *USFWS* U.S. Fish and Wildlife Service

**Table 4:** Correlation values between hydrologic signatures and variables

Variable Type	Variable	Flashiness Index	Flashiness (wet season)	MAX 30/area	(Q10-Q95)/area	DryMonth/area	Baseflow index

Climate	Precipitation	0.06	0.01	0.86	0.87	0.68	0.16
	Evapotranspiration	0.43	0.32	0.18	0.14	−0.15	−0.47
	Aridity index	−0.02	−0.04	−0.77	−0.80	−0.86	−0.42
	Water demand	−0.03	−0.02	0.78	0.83	0.82	0.41
	Precipitation seasonality	0.17	0.26	0.01	−0.04	0.21	0.20
	Precipitation CV	0.18	0.17	−0.59	−0.65	−0.85	−0.59
	Max monthly precipitation	0.30	0.25	0.85	0.82	0.48	−0.10
	Temperature seasonality	−0.29	−0.18	−0.30	−0.28	−0.05	0.24
	Temperature CV	−0.38	−0.29	−0.35	−0.30	−0.08	0.28

Land cover	Forest	−0.14	−0.17	0.28	0.32	0.18	0.15
	Developed	0.22	0.18	0.62	0.60	0.62	0.18
	Cultivated crops	−0.16	−0.13	0.03	0.06	0.30	0.27
	Stream density	0.36	0.29	0.37	0.35	−0.06	−0.33

Soil and Geology	Clay fraction	0.40	0.37	0.25	0.15	−0.12	−0.44
	Sand fraction	−0.23	−0.27	−0.32	−0.26	−0.07	0.16
	Silt fraction	0.04	0.10	0.18	0.17	0.12	0.06
	Depth to bedrock	−0.29	−0.30	0.14	0.18	0.32	0.20
	Water table depth	0.12	0.13	−0.54	−0.55	−0.45	−0.09
	Geologic permeability	−0.42	−0.40	−0.25	−0.20	0.16	0.43

Topography	Slope	0.13	0.13	−0.23	−0.22	−0.27	0.00
	Elevation range	0.12	0.03	0.24	0.24	0.14	−0.04
	Topographic diversity	0.11	0.11	−0.17	−0.15	−0.20	0.04

Inundation Dynamics	Temporarily flooded, FP	0.27	0.23	0.42	0.40	0.24	−0.05
	Temporarily inundated, NFP	−0.06	−0.03	0.49	0.51	0.58	0.30
	Seasonally inundated, FP	−0.12	−0.15	0.66	0.69	0.59	0.15
	Seasonally inundated, NFP	−0.21	−0.19	0.36	0.37	0.39	0.14
	SP+P inundated, FP	−0.44	−0.46	0.24	0.33	0.33	0.14
	SP+P, inundated, NFP	−0.34	−0.32	0.13	0.11	0.13	0.04
	Total inundation, FP	−0.12	−0.15	0.60	0.63	0.52	0.12
	Total inundation, NFP	−0.19	−0.17	0.37	0.37	0.41	0.17
	Proport. Seasonally inundated, FP	−0.17	−0.20	0.41	0.46	0.36	0.17
	Proport. Seasonally inundated, NFP	−0.20	−0.16	0.06	0.04	0.11	0.09

Wetland	GIW	−0.31	−0.29	0.07	0.08	0.13	0.04
	Prop. of wetland area that is GIW	−0.08	−0.05	0.08	0.03	0.06	0.01
	Floodplain	−0.02	−0.07	0.49	0.51	0.39	0.00
	NWI wetlands	−0.44	−0.44	0.12	0.19	0.28	0.19

Significant (*p* < 0.01) correlations, after Bonferroni correction was applied, are shown in shaded gray

*CV* coefficient of variation, *FP* floodplain, *NFP* non-floodplain, *Prop* proportion, *MAX* maximum, *SP + P* semi-permanent and permanent, *Proport.* proportion, *GIW* geographically isolated wetlands, *NWI* National Wetland Inventory

**Table 5:** Model statistics for each hydrologic signature and version of the model including (1) climate variables only (M_Climate_), (2) climate, inundation and wetland variables (M_Climate+Water_), and (3) all variables (M_All_)

Signature	Model	R^2^	R^2^ adj	RMSE	MSE	AIC	Trees	Variable count

Flashiness index	M_Climate_	0.57	0.54	0.236	0.056	−82.3	1000	4
	M_Climate+Water_	0.58	0.54	0.233	0.054	−81.1	700	5
	M_All_	0.60	0.57	0.226	0.051	−85.0	700	4
Flashiness index (wet season)	M_Climate_	0.48	0.44	0.272	0.074	−71.5	500	4
	M_Climate+Water_	0.49	0.44	0.269	0.072	−74.2	1000	5
	M_All_	0.52	0.46	0.261	0.068	−76.0	300	6
MAX30/ area	M_Climate_	0.65	0.63	0.475	0.202	−40.0	1000	3
	M_Climate+Water_	0.69	0.66	0.449	0.201	−42.1	1000	4
	M_All_	0.69	0.66	0.445	0.192	−45.6	700	5
(Q10-Q95)/area	M_Climate_	0.75	0.73	0.007	0.000047	−301.8	1000	3
	M_Climate+Water_	0.77	0.74	0.007	0.000044	−305.5	700	4
	M_All_	0.78	0.76	0.006	0.000041	−310.0	700	6
DryMonth/ area	M_Climate_	0.83	0.82	0.001	0.000001	−416.4	700	4
	M_Climate+Water_	0.87	0.85	0.001	0.000001	−421.1	300	5
	M_All_	0.87	0.85	0.001	0.000001	−421.1	300	5
Baseflow index	M_Climate_	0.58	0.55	0.117	0.014	−126.0	300	4
	M_Climate+Water_	0.65	0.62	0.107	0.012	−131.6	1000	4
	M_All_	0.66	0.63	0.105	0.011	−133.1	700	5

Maximum tree depth was 4 for all models, except for (Q10-Q95)/area M_Climate_ which had a maximum tree depth of 3

*RMSE* root mean square error, *adj* adjusted, *MSE* mean square error, *AIC* Akaike information criterion

## Data Availability

The surface water data produced for this analysis are published and available (Vanderhoof et al. 2024b).

## Appendix

See Tables 6, 7, 8, 9 and 10.

**Table 6:** The 72 U.S. Geological survey gages and watersheds included in the analysis

Gage ID	Site ID	U.S. State(s)	Area (km^2^)	NHD Density (m km^2^)	NWI (% area)	PDSI (min, %)	PDSI (max, %)	PDSI (median, %)	Primary land cover

01491000	MD1	MD, DE	292	2030.7	28.6	32.6	100.0	66.5	CC (47%)
01578475	MD2	MD, PA	458	1069.2	2.6	6.1	100.0	72.4	CC (43%)
01580520	MD3	MD, PA	425	1130.1	2.1	8.9	100.0	67.4	DF (30%)
01594440	MD4	MD	907	1571.9	6.4	16.7	100.0	64.4	D (36%)
01643000	MD5	MD, PA	2112	1394.3	3.0	4.2	100.0	57.5	HP (27%)
02049500	VA1	VA	1583	1497.8	15.7	33.0	100.0	79.3	EF (29%)
02131500	SC1	SC, NC	1720	1451.6	10.3	18.3	100.0	53.7	EF (26%)
02135000	SC2	SC, NC	7256	1628.6	27.2	8.5	100.0	78.0	WW (31%), CC (31%)
02136000	SC3	SC	3211	1738.0	27.0	17.6	100.0	75.2	CC (32%), WW (31%)
02175000	SC4	SC	7077	1163.0	17.3	27.1	98.0	75.5	EF (25%), WW (24%)
02198000	GA1	GA	1676	1365.2	12.0	19.4	96.6	61.1	EF (26%)
02202500	GA2	GA	6887	1249.8	16.8	21.1	97.7	60.2	EF (26%)
05056000	ND1	ND	4862	283.9	10.6	1.2	100.0	56.3	CC (52%)
05057200	ND2	ND	1897	259.2	11.6	0.0	100.0	65.0	CC (67%)
05062500	MN1	MN	2407	745.9	23.9	3.4	100.0	58.6	CC (39%)
05066500	ND3	ND	3218	774.1	6.9	0.3	100.0	63.4	CC (81%)
05078500	MN2	MN	3518	862.3	23.5	1.2	100.0	54.5	CC (48%)
05090000	ND4	ND	1742	1068.9	3.7	1.5	100.0	51.0	CC (73%)
05123400	ND5	ND	3206	515.6	12.2	1.0	97.8	48.8	CC (48%)
05131500	MN3	MN	4384	608.9	42.4	4.5	100.0	84.4	WW (49%)
05132000	MN4	MN	3895	537.3	48.7	5.6	100.0	71.1	WW (49%)
05244000	MN5	MN	2683	471.2	23.8	0.9	100.0	52.3	DF (27%)
05300000	MN6	MN, SD	2468	1286.4	11.5	11.6	100.0	66.6	CC (68%)
05304500	MN7	MN	4899	733.6	17.0	4.8	100.0	62.5	CC (66%)
05313500	MN8	MN, SD	1801	1129.0	8.8	8.5	100.0	58.8	CC (80%)
05336700	MN9	MN	2252	676.5	34.1	17.0	100.0	87.8	WW (34%)
05388250	IA1	IA, MN	2010	1548.4	2.7	9.2	100.0	76.1	CC (61%)
05412500	IA2	IA	3858	1414.9	2.4	6.9	100.0	81.6	CC (66%)
05418500	IA3	IA	4019	1452.5	2.1	6.3	100.0	70.8	CC (69%)
05422000	IA4	IA, MN	6049	1248.5	4.6	5.9	99.7	70.2	CC (79%)
05434500	WI1	WI, IL	2677	1618.6	3.0	5.1	100.0	71.9	CC (44%)
05447500	IL1	IL	2576	1115.6	1.9	20.7	100.0	74.9	CC (85%)
06018500	MT1	MT	9373	1628.9	3.9	0.3	89.3	50.9	SS (47%)
06052500	MT2	MT, WY	4634	1376.2	2.9	1.2	97.4	61.8	EF (47%)
06076690	MT3	MT	2189	1695.3	4.3	1.4	98.1	62.7	H (35%)
06468170	ND6	ND	2809	302.6	7.4	1.0	100.0	66.3	CC (67%)
06471200	ND7	ND, SD	1869	627.2	11.2	1.2	100.0	70.8	CC (62%)
06479525	SD1	SD	2467	947.8	9.8	19.3	100.0	67.4	CC (59%)
06481500	SD2	SD	1604	1102.0	8.7	8.8	100.0	62.0	CC (72%)
06815000	NE1	NE, KS	3473	1688.2	1.8	4.1	99.2	52.8	CC (54%)
06821190	MO1	MO, IA	6179	1925.6	4.8	11.3	99.0	56.6	CC (50%)
06908000	MO2	MO	2895	1737.9	4.2	3.5	90.4	51.9	HP (38%)
06916600	KS2	KS, MO	8387	1685.9	3.8	12.3	100.0	57.5	HP (37%)
06918060	MO3	MO, KS	2773	1669.2	5.4	4.7	100.0	57.0	HP (56%)
06928000	MO4	MO	3275	1538.7	1.8	12.8	100.0	79.9	DF (45%), HP (43%)
07047950	AR1	AR	1985	1864.2	12.5	20.2	100.0	82.5	CC (73%)
07169500	KS3	KS	2098	1781.3	2.9	4.9	100.0	62.0	H (595)
07288500	MS1	MS	2009	1809.9	9.8	7.1	97.9	55.9	CCs (82%)
07290000	MS2	MS	7124	2565.5	10.0	13.3	100.0	74.8	EF (19%), MF (19%)
07346070	TX1	TX	1809	2010.3	9.3	6.5	100.0	70.4	HP (27%)
07363500	AR2	AR	5429	1762.5	3.0	28.8	99.1	83.0	EF (40%)
07364200	LA1	AR, LA	3138	1507.9	14.6	22.8	100.0	79.5	CC (31%)
08033500	TX2	TX	9406	1712.0	8.0	3.2	99.9	64.9	EF (29%)
08068090	TX4	TX	2539	1695.0	9.9	10.9	100.0	71.4	EF (32%)
08110000	TX5	TX	2616	1630.0	4.8	8.9	100.0	73.8	HP (55%)
08117500	TX6	TX	1869	1085.4	5.6	6.4	98.6	64.8	HP (43%)
08164000	TX7	TX	2124	1435.4	2.1	8.8	94.1	52.3	HP (59%)
09439000	AZ1	AZ, NM	9279	1679.3	1.2	1.2	98.1	40.2	SS (45%)
09485700	AZ2	AZ	2238	2347.0	2.1	0.0	95.4	48.3	SS (64%)
09487000	AZ3	AZ	2028	3229.6	2.3	0.0	87.7	42.4	SS (79%)
09512800	AZ4	AZ	2876	1639.6	1.3	0.1	88.1	47.1	SS (68%)
09517000	AZ5	AZ	3967	1664.7	1.7	0.2	90.8	50.6	SS (81%)
09537500	AZ6	AZ	2912	1392.5	1.1	0.0	96.6	46.0	SS (67%)
11348500	CA1	CA	3884	1469.4	8.0	0.0	84.1	55.6	SS (50%)
11376000	CA2	CA	2313	2450.2	1.9	0.0	89.1	29.9	SS (56%)
11473900	CA3	CA	1925	4181.6	1.2	0.0	88.2	35.5	EF (45%)
11501000	OR1	OR	4121	1028.4	8.2	0.0	83.3	43.4	EF (55%)
11517500	CA4	CA	2047	1495.8	5.6	0.0	94.6	17.6	EF (37%)
11519500	CA5	CA	1714	2381.7	3.8	0.0	97.6	26.3	EF (46%)
12324680	MT4	MT	4590	1287.2	3.5	1.4	97.7	46.4	EF (45%)
13302005	ID1	ID	2143	1615.5	1.2	0.5	97.8	51.2	SS (76%)
13305000	ID2	ID	2412	1443.0	1.3	0.5	93.6	48.6	SS (59%)
All (median)	~	~	2647	1461.0	5.6	5.0	100.0	62.0	~

The 2016–2023 period is shown relative to the Palmer Drought Severity Index (PDSI, 1980–2021)

*NHD* National hydrographic dataset, *NWI* National Wetland Inventory, *CC* cultivated crops, *DF* deciduous forest, *D* developed, *HP* hay/pasture, *EF* evergreen forest, *WW* woody wetlands, *MF* mixed forest, *SS* shrub/scrub, *H* herbaceous

**Table 7:** Thresholds selected from 5-year Sentinel-1 (S1) and Sentinel-2 (S2) based surface water percentiles to account for variable accuracy between sites, sensors, and classes (open water (OW) compared to vegetated water (VW)

Site ID	S1 OW (%)	S1 VW (%)	S2 OW (%)	S2 VW (%)	Site ID	S1 OW (%)	S1 VW (%)	S2 OW (%)	S2 VW (%)

AR1	15	30	15	30	MN7	10	35	5	25
AR2	5	20	10	25	MN8	10	25	5	15
AZ1	10	5	15	10	MN9	5	25	5	25
AZ2	5	15	10	15	MO1	10	20	10	20
AZ3	5	10	10	15	MO2	5	15	15	25
AZ4	5	20	15	20	MO3	5	30	10	30
AZ5	5	20	20	15	MO4	10	15	10	35
AZ6	10	15	10	~	MS1	10	30	10	35
CA1	5	15	10	20	MS2	5	10	5	30
CA2	10	10	15	15	MT1	25	~	10	30
CA3	10	~	15	15	MT2	25	~	15	40
CA4	10	15	20	20	MT3	30	~	10	40
CA5	10	10	20	15	MT4	30	~	10	30
GA1	5	5	5	20	ND1	15	20	5	10
GA2	5	10	10	15	ND2	20	20	10	20
IA1	~	15	10	15	ND3	15	~	5	20
IA2	~	10	10	20	ND4	15	35	5	30
IA3	10	10	10	20	ND5	20	30	5	25
IA4	10	20	10	20	ND6	15	30	5	25
ID1	30	~	15	35	ND7	20	30	5	20
ID2	30	~	20	35	NE1	15	15	10	20
IL1	10	30	10	15	OR1	10	20	25	25
KS2	10	20	10	30	SC1	5	20	10	35
KS3	~	15	10	20	SC2	5	25	5	25
LA1	10	25	15	35	SC3	5	30	5	30
MD1	5	~	10	20	SC4	5	30	10	35
MD2	5	15	10	20	SD1	10	25	5	25
MD3	5	10	10	15	SD2	15	25	5	25
MD4	5	10	10	15	TX1	5	10	10	30
MD5	10	30	15	~	TX2	5	30	10	35
MN1	15	20	5	20	TX4	5	30	10	35
MN2	10	20	5	30	TX5	5	20	10	~
MN3	10	30	10	30	TX6	10	~	10	35
MN4	5	30	5	30	TX7	5	35	10	30
MN5	10	30	5	25	VA1	5	30	10	45
MN6	15	25	5	20	WI1	~	15	10	20

~ indicates that this output was excluded from the allowable water mask

**Table 8:** Hydrologic signatures by watershed

Region	ID	Gage	Flashiness Index	Flashiness index (wet season)	MAX30/area	(Q10-Q95)/area	Dry Month/area	Baseflow index

East	**East**		**−0.74**	**−0.78**	**1.37**	**0.023**	**0.0065**	**0.38**
	MD1	01491000	−0.48	−0.45	2.16	0.034	0.0072	0.28
	MD2	01578475	−0.44	−0.43	1.52	0.024	0.0105	0.55
	MD3	01580520	−0.52	−0.64	1.45	0.024	0.0112	0.54
	MD4	01594440	−0.43	−0.42	1.38	0.021	0.0089	0.49
	MD5	01643000	−0.35	−0.40	1.98	0.028	0.0058	0.24
	VA1	02049500	−0.87	−1.01	1.27	0.028	0.0060	0.36
	SC1	02131500	−0.66	−0.64	1.29	0.022	0.0059	0.39
	SC2	02135000	−1.05	−1.07	1.55	0.025	0.0055	0.35
	SC3	02136000	−0.91	−1.04	1.22	0.023	0.0039	0.28
	SC4	02175000	−1.13	−1.20	0.89	0.017	0.0055	0.44
	GA1	02198000	−0.90	−0.95	0.86	0.016	0.0043	0.37
	GA2	02202500	−1.09	−1.17	0.92	0.017	0.0030	0.24

Gulf Coast	**Gulf Coast**		**−0.79**	**−0.83**	**1.88**	**0.032**	**0.0026**	**0.09**
	AR1	07047950	−0.99	−1.01	3.48	0.050	0.0057	0.18
	MS1	07288500	−0.79	−0.90	2.23	0.056	0.0035	0.04
	MS2	07290000	−0.85	−0.93	2.22	0.046	0.0030	0.10
	TX1	07346070	−0.74	−0.71	1.64	0.025	0.0006	0.02
	AR2	07363500	−0.82	−0.86	2.46	0.050	0.0024	0.05
	LA1	07364200	−1.45	−1.58	1.37	0.044	0.0030	0.16
	TX2	08033500	−0.94	−1.01	1.19	0.024	0.0027	0.08
	TX4	08068090	−0.35	−0.31	2.30	0.016	0.0022	0.09
	TX5	08110000	−1.00	−1.02	0.54	0.020	0.0024	0.08
	TX6	08117500	−0.51	−0.59	2.10	0.021	0.0019	0.08
	TX7	08164000	−0.21	−0.23	1.13	0.003	0.0010	0.07

Midwest	**Midwest**		**−0.62**	**−0.60**	**1.43**	**0.021**	**0.0042**	**0.28**
	IA1	05388250	−0.78	−0.68	1.51	0.025	0.0083	0.47
	IA2	05412500	−0.73	−0.62	1.53	0.024	0.0066	0.37
	IA3	05418500	−0.80	−0.69	1.11	0.016	0.0077	0.59
	IA4	05422000	−0.99	−1.06	1.14	0.023	0.0060	0.41
	WI1	05434500	−1.12	−1.01	0.96	0.014	0.0094	0.70
	IL1	05447500	−0.79	−0.78	1.03	0.018	0.0055	0.38
	NE1	06815000	−0.25	−0.21	0.96	0.007	0.0013	0.24
	MO1	06821190	−0.52	−0.55	1.14	0.016	0.0018	0.19
	MO2	06908000	−0.40	−0.44	1.61	0.022	0.0010	0.05
	KS2	06916600	−0.55	−0.60	1.48	0.023	0.0013	0.09
	MO3	06918060	−0.39	−0.45	2.13	0.030	0.0020	0.06
	MO4	06928000	−0.38	−0.34	2.24	0.026	0.0024	0.10
	KS3	07169500	−0.42	−0.36	1.69	0.032	0.0015	0.06

North-Central	**North-Central**		**−0.93**	**−0.93**	**0.52**	**0.008**	**0.0016**	**0.19**
	ND1	05056000	−1.04	−0.98	0.11	0.002	0.0005	0.08
	ND2	05057200	−0.83	−0.88	0.21	0.004	0.0004	0.07
	MN1	05062500	−0.94	−0.92	0.48	0.007	0.0014	0.24
	ND3	05066500	−0.76	−0.79	0.54	0.006	0.0007	0.09
	MN2	05078500	−0.81	−0.77	0.54	0.006	0.0011	0.23
	ND4	05090000	−0.78	−0.82	0.34	0.004	0.0004	0.05
	ND5	05123400	−1.09	−1.11	0.10	0.002	0.0001	0.06
	MN3	05131500	−0.90	−0.86	1.15	0.018	0.0028	0.19
	MN4	05132000	−1.01	−0.95	0.77	0.013	0.0020	0.27
	MN5	05244000	−1.45	−1.46	0.31	0.006	0.0038	0.68
	MN6	05300000	−0.99	−0.96	0.65	0.011	0.0019	0.21
	MN7	05304500	−1.16	−1.14	0.46	0.010	0.0029	0.34
	MN8	05313500	−0.90	−0.89	0.83	0.015	0.0025	0.18

Region	ID	Gage	Flashiness Index	Flashiness index (wet season)	MAX30/area	(Q10-Q95)/area	Dry Month/area	Baseflow index

	MN9	05336700	−0.77	−0.78	1.69	0.027	0.0055	0.25
	ND6	06468170	−0.93	−0.93	0.18	0.003	0.0001	0.04
	ND7	06471200	−0.68	−0.64	0.24	0.002	0.0002	0.09
	SD1	06479525	−1.00	−1.09	0.23	0.005	0.0010	0.22
	SD2	06481500	−0.73	−0.73	0.48	0.009	0.0016	0.18

Southwest	**Southwest**		**−0.12**	**−0.16**	**0.06**	**<0.001**	**<0.0001**	**0.01**
	AZ1	09439000	−0.61	−0.83	0.09	0.001	0.0000	0.03
	AZ2	09485700	0.07	0.12	0.08	0.000	0.0000	0.00
	AZ3	09487000	0.23	0.23	0.01	0.000	0.0000	0.00
	AZ4	09512800	−0.08	−0.09	0.16	0.001	0.0000	0.00
	AZ5	09517000	−0.30	−0.34	0.02	0.000	0.0001	0.05
	AZ6	09537500	−0.02	−0.03	0.01	0.000	0.0000	0.00

West	**West**		**−1.03**	**−1.09**	**0.67**	**0.012**	**0.0009**	**0.26**
	MT1	06018500	−1.23	−1.41	0.04	0.001	0.0004	0.44
	MT2	06052500	−1.18	−1.06	0.69	0.013	0.0022	0.32
	MT3	06076690	−1.04	−1.01	0.18	0.004	0.0007	0.32
	CA1	11348500	−0.70	−0.77	0.22	0.004	0.0001	0.02
	CA2	11376000	−0.51	−0.69	1.62	0.023	0.0006	0.06
	CA3	11473900	−0.51	−0.69	3.31	0.051	0.0003	0.01
	OR1	11501000	−1.25	−1.24	0.31	0.005	0.0011	0.35
	CA4	11517500	−1.13	−1.27	0.14	0.003	0.0005	0.21
	CA5	11519500	−0.82	−0.95	0.92	0.023	0.0003	0.03
	MT4	12324680	−1.16	−1.07	0.33	0.006	0.0015	0.38
	ID1	13302005	−1.63	−1.89	0.12	0.002	0.0019	0.61
	ID2	13305000	−1.22	−1.08	0.20	0.003	0.0012	0.40

The blue to red shading reflects the high to low values for each signature. The bold values indicate the average values for the watersheds within each region.

**Table 9:** Correlation values between remotely sensed surface water variables and other independent variables.

Variable Type	Variable	Temp. flooded, FP	Temp. inun., NFP	Seas. inun., FP	Seas. inun., NFP	SP+P inun., FP	SP+P inun., NFP	Total inun., FP	Total inun., NFP	Prop. seas. inun., FP	Prop. seas. inun., NFP

Climate	Precipitation	0.39	0.52	0.75	0.44	0.41	0.21	0.69	0.45	0.47	0.06
	Evapotranspiration	0.40	−0.12	0.19	−0.22	−0.10	−0.27	0.19	−0.23	0.22	−0.44
	Aridity index	−0.27	−0.69	−0.67	−0.55	−0.40	−0.26	−0.61	−0.59	−0.28	−0.20
	Water demand	0.22	0.61	0.61	0.46	0.34	0.16	0.53	0.50	0.31	0.16
	Precipitation seasonality	0.03	0.19	0.06	0.29	0.03	0.09	0.11	0.26	−0.20	0.32
	Precipitation CV	−0.20	−0.52	−0.64	−0.45	−0.38	−0.25	−0.55	−0.46	−0.41	−0.17
	Max monthly precipitation	0.51	0.44	0.63	0.33	0.26	0.09	0.63	0.34	0.34	−0.06
	Temperature seasonality	−0.37	0.02	−0.25	0.19	0.06	0.23	−0.21	0.19	−0.31	0.46
	Temperature CV	−0.44	−0.01	−0.32	0.14	0.05	0.23	−0.28	0.15	−0.32	0.43

Land Cover	Forest	0.00	0.30	0.10	−0.02	0.06	0.00	0.04	0.04	0.20	−0.15
	Developed	0.39	0.37	0.63	0.28	0.28	0.04	0.58	0.28	0.44	−0.04
	Cultivated crops	0.07	0.05	0.21	0.28	0.17	0.16	0.23	0.25	0.04	0.30
	Stream density	0.43	−0.11	0.13	−0.32	−0.24	−0.48	0.13	−0.32	0.32	−0.45

Soil and Geology	Clay fraction	0.39	−0.01	0.27	0.00	0.00	−0.10	0.27	−0.06	0.20	−0.14
	Sand fraction	−0.35	0.05	−0.17	0.08	0.10	0.22	−0.18	0.09	−0.25	0.11
	Silt Fraction	0.22	−0.06	0.02	−0.12	−0.15	−0.25	0.04	−0.11	0.19	−0.07
	Depth to bedrock	−0.12	0.33	0.49	0.71	0.66	0.69	0.51	0.68	−0.03	0.52
	Water table depth	−0.18	−0.51	−0.68	−0.78	−0.67	−0.64	−0.72	−0.73	−0.12	−0.47
	Geologic permeability	−0.36	0.16	−0.06	0.21	0.18	0.31	−0.09	0.21	−0.17	0.27

Topography	Slope	0.02	−0.30	−0.55	−0.77	−0.63	−0.76	−0.56	−0.71	0.05	−0.59
	Elevation range	0.21	0.02	0.12	−0.22	−0.13	−0.23	0.04	−0.18	0.24	−0.35
	Topographic diversity	0.02	−0.22	−0.50	−0.71	−0.57	−0.70	−0.51	−0.65	0.05	−0.58

Wetland	GIW	−0.27	0.32	0.37	0.80	0.73	0.89	0.40	0.76	−0.21	0.73
	Prop. of wetland area that is GIW	−0.09	0.14	0.26	0.55	0.38	0.62	0.29	0.50	−0.12	0.59
	Floodplain	0.64	0.28	0.84	0.36	0.55	0.19	0.92	0.30	0.57	−0.17
	NWI wetlands	−0.27	0.48	0.45	0.81	0.86	0.85	0.46	0.80	−0.13	0.60

Significant (*p* < 0.01) correlations, after Bonferroni correction has been applied, are shown shaded in gray. Correlations between surface water variables ranged from 0 to 0.98 with a median correlation of 0.35

*CV* coefficient of variation, *FP* floodplain, *NFP* non-floodplain, *temp* temporarily, *seas* seasonally, *SP + P* semi-permanent to permanent, *inun* inundation, *Prop.* proportion, *GIW* Geographically Isolated Wetlands

**Table 10:** Correlation values between independent variables.

Variable	Precip.	ET	Aridity index	Water demand	Precip. season.	Precip CV	Max. monthly precip.	Temp. season.	Temp. CV	Forest	Dev.	Ag.	Stream density

Precipitation (precip.)	1.00	0.32	−0.79	0.82	−0.11	−0.73	0.88	−0.44	−0.49	0.30	0.77	0.07	0.33
Evapotranspiration (ET)	0.32	1.00	0.13	−0.13	−0.44	0.04	0.45	−0.90	−0.95	0.19	0.32	−0.44	0.63
Aridity index	−0.79	0.13	1.00	−0.95	−0.24	0.80	−0.64	0.04	0.08	−0.20	−0.67	−0.27	−0.02
Water demand	0.82	−0.13	−0.95	1.00	0.16	−0.79	0.66	−0.04	−0.07	0.17	0.63	0.28	0.10
Precip. season.	−0.11	−0.44	−0.24	0.16	1.00	0.02	0.00	0.59	0.43	−0.49	0.10	0.55	−0.38
Precipitation CV	−0.73	0.04	0.80	−0.79	0.02	1.00	−0.46	0.19	0.18	−0.28	−0.63	−0.13	−0.05
Max. monthly precip.	0.88	0.45	−0.64	0.66	0.00	−0.46	1.00	−0.47	−0.56	0.18	0.67	−0.03	0.46
Temp. season.	−0.44	−0.90	0.04	−0.04	0.59	0.19	−0.47	1.00	0.96	−0.42	−0.34	0.55	−0.62
Temperature CV	−0.49	−0.95	0.08	−0.07	0.43	0.18	−0.56	0.96	1.00	−0.30	−0.44	0.46	−0.64
Forest	0.30	0.19	−0.20	0.17	−0.49	−0.28	0.18	−0.42	−0.30	1.00	0.11	−0.68	0.22
Developed (Dev.)	0.77	0.32	−0.67	0.63	0.10	−0.63	0.67	−0.34	−0.44	0.11	1.00	0.18	0.24
Cultivated crops (Ag.)	0.07	−0.44	−0.27	0.28	0.55	−0.13	−0.03	0.55	0.46	−0.68	0.18	1.00	−0.40
Stream density	0.33	0.63	−0.02	0.10	−0.38	−0.05	0.46	−0.62	−0.64	0.22	0.24	−0.40	1.00
Clay fraction	0.18	0.37	−0.02	−0.01	0.21	0.13	0.36	−0.18	−0.32	−0.14	0.12	−0.02	0.26
Sand fraction	−0.16	0.12	0.21	−0.27	−0.36	−0.03	−0.29	−0.29	−0.17	0.38	−0.12	−0.47	−0.12
Silt fraction	0.04	−0.36	−0.20	0.28	0.38	0.00	0.10	0.48	0.40	−0.36	0.05	0.53	0.00
Depth to bedrock	0.33	−0.23	−0.41	0.35	0.26	−0.35	0.16	0.24	0.21	−0.26	0.30	0.55	−0.43
Water table depth	−0.59	0.13	0.65	−0.58	−0.27	0.52	−0.49	−0.11	−0.05	0.05	−0.42	−0.37	0.10
Geological permeability	−0.13	−0.20	0.04	−0.10	−0.17	−0.19	−0.31	−0.01	0.09	0.29	−0.10	−0.24	−0.31
Slope	−0.31	0.20	0.41	−0.32	−0.34	0.26	−0.24	−0.24	−0.17	0.42	−0.26	−0.56	0.41
Elevation z-score	0.39	0.66	−0.08	0.11	−0.62	−0.24	0.34	−0.79	−0.74	0.44	0.37	−0.45	0.49
Topographic diversity	−0.25	0.22	0.34	−0.26	−0.41	0.18	−0.20	−0.31	−0.22	0.52	−0.23	−0.65	0.42
GIW	0.13	−0.39	−0.26	0.16	0.33	−0.14	0.04	0.43	0.39	−0.24	0.10	0.46	−0.58
GIW wetland proportion	0.04	−0.43	−0.17	0.08	0.51	0.00	0.05	0.55	0.46	−0.39	0.10	0.60	−0.58
Floodplain (FP)	0.60	0.35	−0.47	0.40	0.11	−0.40	0.61	−0.34	−0.42	0.03	0.52	0.12	0.19
NWI wetlands	0.26	−0.25	−0.37	0.27	0.10	−0.34	0.08	0.19	0.21	0.02	0.18	0.24	−0.37

Variable	Clay	Sand	Silt	Depth	Water	Geologic	Slope	Elev. z-	Topo.	GIW	GIW	FP	NWI

Precipitation	0.18	−0.16	0.04	0.33	−0.59	−0.13	−0.31	0.39	−0.25	0.13	0.04	0.60	0.26
Evapotranspiration (ET)	0.37	0.12	−0.36	−0.23	0.13	−0.20	0.20	0.66	0.22	−0.39	−0.43	0.35	−0.25
Aridity index	−0.02	0.21	−0.20	−0.41	0.65	0.04	0.41	−0.08	0.34	−0.26	−0.17	−0.47	−0.37
Water demand	−0.01	−0.27	0.28	0.35	−0.58	−0.10	−0.32	0.11	−0.26	0.16	0.08	0.40	0.27
Precip. season.	0.21	−0.36	0.38	0.26	−0.27	−0.17	−0.34	−0.62	−0.41	0.33	0.51	0.11	0.10
Precipitation CV	0.13	−0.03	0.00	−0.35	0.52	−0.19	0.26	−0.24	0.18	−0.14	0.00	−0.40	−0.34
Max. monthly precip.	0.36	−0.29	0.10	0.16	−0.49	−0.31	−0.24	0.34	−0.20	0.04	0.05	0.61	0.08
Temp. season.	−0.18	−0.29	0.48	0.24	−0.11	−0.01	−0.24	−0.79	−0.31	0.43	0.55	−0.34	0.19
Temperature CV	−0.32	−0.17	0.40	0.21	−0.05	0.09	−0.17	−0.74	−0.22	0.39	0.46	−0.42	0.21
Forest	−0.14	0.38	−0.36	−0.26	0.05	0.29	0.42	0.44	0.52	−0.24	−0.39	0.03	0.02
Developed (Dev.)	0.12	−0.12	0.05	0.30	−0.42	−0.10	−0.26	0.37	−0.23	0.10	0.10	0.52	0.18
Cultivated crops (Ag.)	−0.02	−0.47	0.53	0.55	−0.37	−0.24	−0.56	−0.45	−0.65	0.46	0.60	0.12	0.24
Stream density	0.26	−0.12	0.00	−0.43	0.10	−0.31	0.41	0.49	0.42	−0.58	−0.58	0.19	−0.37
Clay fraction	1.00	−0.56	0.17	−0.09	−0.14	−0.63	−0.08	−0.04	−0.13	−0.06	0.08	0.36	−0.19
Sand fraction	−0.56	1.00	−0.88	0.00	0.12	0.86	0.08	0.34	0.18	0.05	−0.27	−0.19	0.29
Silt fraction	0.17	−0.88	1.00	−0.02	−0.03	−0.68	0.02	−0.41	−0.08	−0.06	0.23	0.02	−0.27
Depth to bedrock	−0.09	0.00	−0.02	1.00	−0.67	0.11	−0.82	−0.26	−0.83	0.78	0.62	0.38	0.72
Water table depth	−0.14	0.12	−0.03	−0.67	1.00	0.00	0.79	0.16	0.72	−0.68	−0.49	−0.58	−0.69
Geologic permeability	−0.63	0.86	−0.68	0.11	0.00	1.00	−0.05	0.12	0.04	0.19	−0.08	−0.18	0.37
Slope	−0.08	0.08	0.02	−0.82	0.79	−0.05	1.00	0.29	0.98	−0.86	−0.71	−0.42	−0.72
Elevation z-score	−0.04	0.34	−0.41	−0.26	0.16	0.12	0.29	1.00	0.36	−0.42	−0.52	0.09	−0.17
Topographic diversity	−0.13	0.18	−0.08	−0.83	0.72	0.04	0.98	0.36	1.00	−0.84	−0.75	−0.38	−0.65
GIW	−0.06	0.05	−0.06	0.78	−0.68	0.19	−0.86	−0.42	−0.84	1.00	0.82	0.23	0.83
GIW wetland proportion	0.08	−0.27	0.23	0.62	−0.49	−0.08	−0.71	−0.52	−0.75	0.82	1.00	0.14	0.42
Floodplain (FP)	0.36	−0.19	0.02	0.38	−0.58	−0.18	−0.42	0.09	−0.38	0.23	0.14	1.00	0.30
NWI wetlands	−0.19	0.29	−0.27	0.72	−0.69	0.37	−0.72	−0.17	−0.65	0.83	0.42	0.30	1.00

Significant (*p* < 0.01) correlations, after Bonferroni correction has been applied, are shown shaded in gray. *CV* coefficient of variation, *season* seasonality, *temp* temperature, *topo divers* topographic diversity, *propor* proportion, *permeab.* permability
